# Predicting the Assembly of the Transmembrane Domains of Viral Channel Forming Proteins and Peptide Drug Screening Using a Docking Approach

**DOI:** 10.3390/biom12121844

**Published:** 2022-12-10

**Authors:** Ta-Chou Huang, Wolfgang B. Fischer

**Affiliations:** Institute of Biophotonics, School of Biomedical Science and Engineering, National Yang Ming Chiao Tung University, No. 155, Sec. 2, Linong St., Taipei 112, Taiwan

**Keywords:** assembly structure prediction, viral membrane proteins, transmembrane domain, docking, peptide screening, molecular dynamics simulations

## Abstract

A de novo assembly algorithm is provided to propose the assembly of bitopic transmembrane domains (TMDs) of membrane proteins. The algorithm is probed using, in particular, viral channel forming proteins (VCPs) such as M2 of influenza A virus, E protein of severe acute respiratory syndrome corona virus (SARS-CoV), 6K of Chikungunya virus (CHIKV), SH of human respiratory syncytial virus (hRSV), and Vpu of human immunodeficiency virus type 2 (HIV-2). The generation of the structures is based on screening a 7-dimensional space. Assembly of the TMDs can be achieved either by simultaneously docking the individual TMDs or via a sequential docking. Scoring based on estimated binding energies (EBEs) of the oligomeric structures is obtained by the tilt to decipher the handedness of the bundles. The bundles match especially well for all-atom models of M2 referring to an experimentally reported tetrameric bundle. Docking of helical poly-peptides to experimental structures of M2 and E protein identifies improving EBEs for positively charged (K,R,H) and aromatic amino acids (F,Y,W). Data are improved when using polypeptides for which the coordinates of the amino acids are adapted to the Cα coordinates of the respective experimentally derived structures of the TMDs of the target proteins.

## 1. Introduction

The prediction of the structure of proteins is a multi-dimensional approach and needs to consider the membrane–protein interactome (MPI) [1]. It includes a series of techniques ranging from potential sequence alignment of the unknown protein with the sequence of known structures to identify eventually common structural patterns, [2] to secondary structure prediction approaches to identify the α-helical transmembrane topology of transmembrane proteins [3,4,5].

The next level of screening is that of orienting the secondary structural elements into their tertiary fold, which also includes rotational screening of the inter-residue orientations [2]. A series of programs, such as Rosetta (rosettacommons.org, accessed on 5 May 2022), AlphaFold (alphafold.ebi.ac.uk, accessed on 6 September 2022), RoseTTAFold (robetta.bakerlab.org, accessed on 24 November 2022) [6] or RaptorX (raptorx.uchicago.edu, accessed on 6 May 2022) are available to propose the fold of, for example, globular proteins to an extremely high level of accuracy, especially when based on neuronal networks [2]. The accuracy of the prediction can reach up to 0.96 Å rmsd_95_ on the Cα atoms covering 95% of the residues with a 95% confidence interval of 0.85–1.16 Å when using, for example, AlphaFold [7]. All approaches have in common that they consider a sequence-based approach supported by extensive screening and comparison of available data. For some of the software, the length of the amino acids (aa’s) of choice should not fall below approximately 25 aa’s (e.g., [6]).

Predicting the fold of TMDs is notoriously difficult, both computationally as well as experimentally. Still, further developments in computational approaches are essential (see, e.g., [8]). In this study, a de novo software, ‘Prediction of Ion Channel Assembly (PICA)’, is presented to generate homo- and hetero-oligomeric assemblies of transmembrane domains (TMDs) and rank them according to a force field (ff)-based scoring. A specialty of the software is to be able to handle any length of a putative TMD identified in the first instant.

Docking approaches in general show limited potency to predict proper poses as in the case of ligand protein interactions (critically reviewed in [9]), and are highly sensitive to the spatial search algorithm as well as the scoring function when dealing with particular proteins (e.g., SARS-CoV-2 protease Mpro in [10]). PICA software can be linked with any newly developed and improved scoring function. It is anticipated that the design of scoring functions is always based on simplified representations of the ‘true’ interactions between molecules. There are options to improve the interactions by using a series of pre-generated protein structures using MD simulations [11] or other computational techniques [9] and with this, also introducing protein dynamics into the search space. In this respect, as PICA generates models and can store them, a whole series of protein assemblies can be used for screening.

The PICA software covers 7 dimensions, distance, rotational angle, tilt, and, in its current extended version, also height of the TMDs, pivot points, and a crossing angle. The quality of the software is assessed based on whether or not the proper handedness of the assembly can be predicted. The software is used conceptually in potential drug screening. A comparison of PICA with available software [12] to predict the assembly of proteins is presented.

The rationale behind the classical docking approach is that it is able to follow and mimic the biological pathway of generating sensible helical TMD assemblies. Bitopic viral channel forming proteins (VCPs) are used as a test case since they are forming homo-oligomers. In the first stage the helical stretch of the TMD needs to be identified using secondary structure prediction programs (see, e.g., [13,14]). Next, a biologically relevant assumption is that the extramembrane domains and the TMDs act independently upon folding; it is assumed that the TMDs will assemble almost independently of the extramembrane domains. This hypothesis might by supported by the fact that in many cases the TMDs are connected with the extramembrane domains via so-called linker regions. These regions are considered to decouple the two domains [15]. Mutations in these regions do not affect the overall mode of action of the protein [16]. On the other hand, they are seen as having their own role in the mechanism of function of membrane proteins [17,18]. Focusing on the TMDs, therefore, the search space can be limited to focus on the TMDs only. This simplifies the search space to an almost 2D approach because many specific orientations should not be available, such as an alignment of a TMD with its helical axis perpendicular to the membrane normal.

The biological pathway includes the assembly of bitopic and polytopic membrane proteins in the translocon [19]. The mechanism is that the TMDs are released from the translocon into the lipid environment [20,21,22]. When considering polytopic membrane proteins, assembling could be assisted by the translocon and its walls [23]. Oligomerization of the membrane proteins is found to be post-translational either within the Golgi complex [24,25]; or, in the outer bacterial membrane, as in the case of bacterial outer-membrane proteins [26]. A relevant feature for a functional relevant assembly is the packing of the helices and with this, the handedness of the bundles [27]. This is especially important when considering the generation of artificial systems [28].

Viral channel forming proteins (VCPs), also called viroporins, are used to probe the quality of PICA. The rational of choosing the VCPs is that many of them are known to be bitopic and structurally characterized [29]. Polytopic VCPs are known and, in several occasions structurally resolved as oligomers via experiments [30,31], or from computational based structural predictions [32]. The role of this type of protein is to change electrochemical gradients across a lipid bilayer either within the virion or within subcellular compartments in the infected cells. In the case of the tetrameric VCP M2 of influenza A, its functioning as a proton channel, triggers a pH change in the interior of the virion, thereby initiating conformational changes of the Influenza A fusion protein haemagglutinin to start the fusion process [33,34]. In addition to ion channel activity, in the case of Vpu, additional functions are reported. Vpu also interacts with other host cell factors, marking them for downregulation by the cell-own degradation machinery [35,36]. For many others, the exact function is yet to be elucidated, albeit isolated studies as mentioned suggest they adopt channel-like behavior.

All VCPs from RNA-viruses chosen in this study are bitopic, with some of them having available experimentally derived structural models: M2 of influenza A [37], E protein of SARS-CoV [29], 6K of Chikungunya virus [38], SH of human respiratory syncytial virus [39], and Vpu of human immunodeficiency virus type 2 [40]. The VCPs are seen as miniaturized channel proteins and taken as a simplified model to elucidate computational algorithms for prediction of the assembled structure of the TMDs. With some of the channels, here M2 and E, already structurally resolved, the assembly software PICA is evaluated and the assembly of structurally unknown assembled TMDs predicted, here, e.g., Vpu, 6K, and SH.

## 2. Materials and Methods

The TMDs of viral channel-forming proteins have either been used and generated from references given in the literature (subscript “e”) or taken from experimental single TMDs or bundle structures from the Protein Data Bank (www.rcsb.org, accessed on 5 May 2022) (subscript “e”). TMDs marked with subscript “p” were generated as ideal helices from applying 20 secondary structure prediction programs to the primary structure and including the amino acids to be part of the TMD if more than 9 out of the 20 programs proposed a helical motif for the particular amino acid. If not mentioned otherwise, the TMD structures were taken as ideal helices (ϕ = −65°, ψ = −39°) indicated by the subscript “i”. Other subscripts such as “28” or “32” indicate the number of amino acids used for modeling the TMD, “t” meaning that a truncated version of the experimentally derived protein is used. Residues which are pointing into the putative pore as shown by experimental structures or assumed based on theoretical considerations are called marker-residues (mr’s) and highlighted in bold and underlined (see below). The subscript “/” means “in combination with”.The residue numbers follow the numbering scheme as in the literature. The sequences used for assembly and MD simulations are as follows:

M2 of influenza A virus:

M2_i_: SDPLVVAASI^32^ IGIL**H^37^**LILWI^42^ L (see also [41,42,43])

M2_e_: SSDPLVVAAS^31^ IIGIL**H^37^**LILW^41^ ILDRL (PDB ID: 1NYJ, left-handed (L) [37])

Marker-residue is H37.

E protein of severe acute respiratory syndrome corona virus (SARS-CoV):

E_p_: LIVNSVLLFL^21^ AFVV**F^26^**LLVTL^31^ AILTAL

E_e_: ETGTLIVNSV^17^ LLFLAFVV**F^26^**L^27^ LVTLAILTAL^37^ RLAAYAANIV^47^

NVSLVKPTVY^57^ VYSRVKNL (PDB ID: 5X29, right-handed (R) [29])

E_t_: ETGTLIVNSV^17^ LLFLAFVV**F^26^**L^27^ LVTLAILTA

Marker-residue is F26.

6K from Chikungunya virus (CHIKV):

6K_p_: LFWLQALIPL^26^ A**A^28^**LIVLCNCL^36^ K (Uniprot sequence Q5XXP3)

Marker-residue is A28 (according to [38]).

SH of human respiratory syncytial virus (hRSV):

SH_p_: FTLI**H^22^**MITTI^27^ I**S^29^**LLIIISIM^37^ IAIL (Uniprot sequence P0DOE5)

SH_e_: MITTI^27^ I**S^29^**LLIIISIM^37^ IAILNKLC (experimental structure but no data

entry [39])

Marker-residues are H22, S29.

Vpu of human immunodeficiency virus type 1 (HIV-1):

Vpu_28_: MQPIPIVAIV^10^ ALVVAIIIAI^20^ VVW**S^24^**IVII

Vpu_32_: MQPIPIVAIV^10^ ALVVAIIIAI^20^ VVW**S^24^**IVIIEY^30^ RK

Vpu_p_: VAIV^10^ ALVVAIIIAI^20^ VVW**S^24^**IVII

(Vpu_28_, Vpu_32_, Vpu_p_: Vpu HV1H2, Uniprot sequence P05919, see also [44])

Vpu_e_: AIV^10^ ALVVAIIIAI^20^ VVW**S^24^**IV (PDB ID: 1PJE [40])

Marker-residue is S24.

### 2.1. Secondary Structure Prediction Programs

The following secondary structure prediction programs were used to predict the TMDs of the proteins denoted by the subscript “p”: CCTOP [45]; DAS (cutoff@1.7) [46] and DAS-TMfilter [47]; HMMTop [48]; MemBrain [49]; Memsat [50] and MEMSAT3 [51]; OCTOPUS [52,53,54] and SPOCTOPUS [52,53]; Philius [55]; Phobius [52] and PolyPhobius [56]; Pro and Prodiv [57]; Scampi [58] and ScampiMsa [58]; SPLIT 4.0 [59]; TMHMM 2.0 [60]; TMMOD [61]; TMpred [62]; TOPOCONS [63]; and PSIPRED 4.0 [64].

### 2.2. Prediction of Ion Channel Assembly (PICA), Assembly and Docking

Assemblies of the TMDs were generated using the in-house developed docking program ‘Prediction of Ion Channel Assembly (PICA)’. The program was encoded in python to generate homo- or hetero-oligomeric models. The TMDs used for docking are either those in which all the atoms of the peptide were considered (AA models), or with AA models after 200 ns of MD simulations (AA-MD models). In addition, the afore mentioned models were also converted into coarse-grained models (CG-models) using the program martinize.py (cgmartini.nl) [65,66].

Two types of screening-resolution were used to explore the conformational space, a large-scaled screening (S1) and a fine-scaled screening (S2). During each screening step, all atoms are positioned according to the protocol. Each conformer was stored in a folder and consequently scored via a potential energy calculation using the Amber99SB-ILDN ff for the AA models and AA-MD models, and the Martini v2.2 ff for the CG-models of the GROMACS suite. The estimated binding energies (EBEs) are calculated on the bases of bonded (bond stretch, angle, and torsional (proper and improper) angles) and non-bonded interactions (short range (Lennard-Jones 12,6) and long range (Coulomb)) as described on both ffs. During energy calculations after the positioning of the TMDs a steepest decent calculation was applied. The calculated potential interaction energies are referred to as EBEs.

The 7-dimensional space was screened including (i) distance, measured from the geometric centers of the TMDs is screened in steps of 0.2 nm (S1) and 0.02 nm (S2); (ii) tilt, screening the range of ±40° (in some cases ±20° and ±30° depending on the models) in steps of ±10° (S1) and ±4° (S2); (iii) rotational angle, screened by rotating the TMD a full 360° around its long axis in steps of ±20° (S1) and ±4° (S2); (iv) tepee-like orientation, screened by a rotation of a single TMD in the range of ±30° around the short-axis perpendicular to the long axis and towards the opposing TMD in steps of ±10° (S1) and ±4° (S2); (v) pivot point, which allows for moving all TMDs around a hinge point towards or away from each other thereby generating wider or narrower tepee-like assemblies, is screened by moving the hinge point in steps of in the range of 0.3 nm (S1) and 0.1 nm (S2); (vi) crossing angle, which is moved along the helix long-axis in steps of 0.3 nm; and (vii) height, in which each TMD is moved along the central axis of the assembly, is screened in the sequential protocol only in steps of 0.4 nm (S1) and 0.1 nm (S2). The protocol allowed for the inclusion of a synchronous screening (s-screening), in which all TMDs were centered along the helical axis around a central pseudo symmetrical axis of C_n_ symmetry (n = number of TMDs) and the dimensions were screened simultaneously by all TMDs. In addition, a sequential or dimeric screening (d-screening) was performed, in which here the assembled group of TMDs of the proteins, here trimeric or tetrameric bundles of M2 and E protein, respectively, was held fixed while only the additional TMD, e.g., here the poly-peptides (pp’s), screens the additional dimension. These oligomeric forms of the M2 and E bundles were obtained by deleting one TMD from each of the original experimental oligomeric bundles and replacing it with the pp. Consequently, the oligomeric bundles were converted into CG models. The pp’s were used either as ideal helices (i) or as models in which the Cα atoms adopt the same coordinates as the experimental structures (Cα).

The pp’s of the 20 amino acids were generated by using the Molecular Operating Environment (MOE) software (v2018, Chemical Computing Group, Tokyo, Japan).

### 2.3. MD Simulations

MD simulations are performed prior to the use of the TMDs in the assembly approach using PICA. The 200 ns MD simulations are considered as to achieve a comprehensive minimization using GROMACS 2020.5 University of Groningen, Groningen, NL) with Amber99SB-ILDN ff containing the lipid parameters from Cordomí et al. [67] and generating models named AA-MD models, in as much the AA models were used for the simulations. The TMDs were embedded into a bilayer patch consisting of 1-palmitoyl-2-oleoyl-*sn*-glycero-3-phosphocholine (POPC) molecules using the manual insertion protocol. Lipids which overlapped with the TMDs were removed. The protein-bilayer system was hydrated with TIP3P water molecules. Sodium- and chloride-ions were added to neutralize the system. Usually the individual systems consisted of 120 POPC lipid molecules, 3655 water molecules and 1 Na^+^/Cl^−^. In the case of E_e_, it consisted of a total of 258 POPC lipid molecules, 10,673 water molecules and 6 Cl^−^ ions as well as M2_i_ with 282 POPC lipid molecules, 10,678 water molecules and 1 Na^+^ ion. Gaps which occurred due to the removal of the lipids were closed by running several steps, including energy minimization steps containing 500 steps of steepest descent and 5000 steps of conjugated gradient to ensure a system without inappropriate geometry or steric clashes and short (1.3 ns) MD simulations under NVT conditions. In these simulations, the temperature of the system rose from 100 K to 200 K and finally to 310 K with the protein restraint with a force constant of k = 1000 kJ mol^−1^ nm^−2^. In the next step, the restraint was released in consequent short simulations at 310 K using force constants of 500, 250 and finally, 0 kJ mol^−1^ nm^−2^. The production run was generated under NPT condition for 200 ns with 1 fs time step length for all samples. In all simulations pressure and temperature were coupled using Berendsen semi-isotropic coupling in x-y directions at 1 bar with 2 ps coupling time and Nose-Hoover coupling with coupling time of 0.1 ps, respectively.

### 2.4. Data Analysis

Microsoft Excel 2016 (Microsoft Corporation, Redmond, WA, USA) and Origin 9.0 (OriginLab, Northampton, MA, USA) are used for data analysis. Data are reported as ‘mean ± SD’ with SD = standard deviation.

### 2.5. Hardware Software

The MD simulation set up and equilibration run were generated on Dell Precision T5810 workstation (Dell Technologies Inc., Round Rock, TX, USA) with 16 Intel Xeon E5-1680 3.2 GHz CPU cores (Intel Corporation, Santa Clara, CA, USA); PICA docking was also performed on a Dell Precision T5820 workstation with 8 Intel Xeon W-2223 3.6 GHz CPU cores including 1 RTX 3080 GPU card, and Acer Veriton M6610 (Acer Inc., Xizhi, New Taipei, Taiwan) with 8 Intel I7-2600 3.4 GHz CPU cores. The production run was performed on the supercomputer Taiwania from National Center for High-performance Computing (NCHC) in Hsinchu, Taiwan, using 64 Intel Xeon Gold 6148 2.4 GHz CPU cores. Visual Molecular Dynamics 1.9.3 (VMD, Urbana-Champaign, IL, USA) was used for data visualization and presentation.

The ColabFold v1.3 software (CF, colabfold.mmseqs.com, accessed on 6 September 2022) [68], which is based on AlphaFold2 and AlphaFold Multimer, was used to generate comparative bundles of tetrameric M2 and pentameric E protein. The software was used in its default mode. The generated bundles were released as ‘relaxed’ PDB structures due to the application of relaxation steps using amber ffs. Further, software GalaxyHomomer (GH, galaxy.seoklab.org/, accessed on 27 October 2022) [69] and IntFold (www.reading.ac.uk/bioinf/IntFOLD/, accessed on 24 October 2022) [70] both were used in their default modes. For all server’s amino acid sequences as for M2_i_ and E_p_ were used.

## 3. Results

### 3.1. Handedness

The conformational space of the TMDs of VCPs is probed by using three representative structural models of the TMDs: (i) AA-models, (ii) AA-MD models (see RMSD values in Supplementary Figure S1) and (iii) CG-models. Respective TMDs are assembled into their relevant oligomeric state using PICA. At first, a S1-screening attempt is undertaken. All models generated are scored on the basis of the EBEs and correlated with the tilt (Supplementary Table S1). This dimension delivers the handedness of the bundles. A tilt of 0° marks straight helices while all the negative/positive tilt angles reflect right/left-handed helices, respectively. The zero tilt is taken as a border-line to separate the two sides of tilt angles. If the EBEs for the structures at zero tilt are not the lowest values, pseudo-minima on either side of the tilt are chosen to identify putative structures.

**Figure 1:**
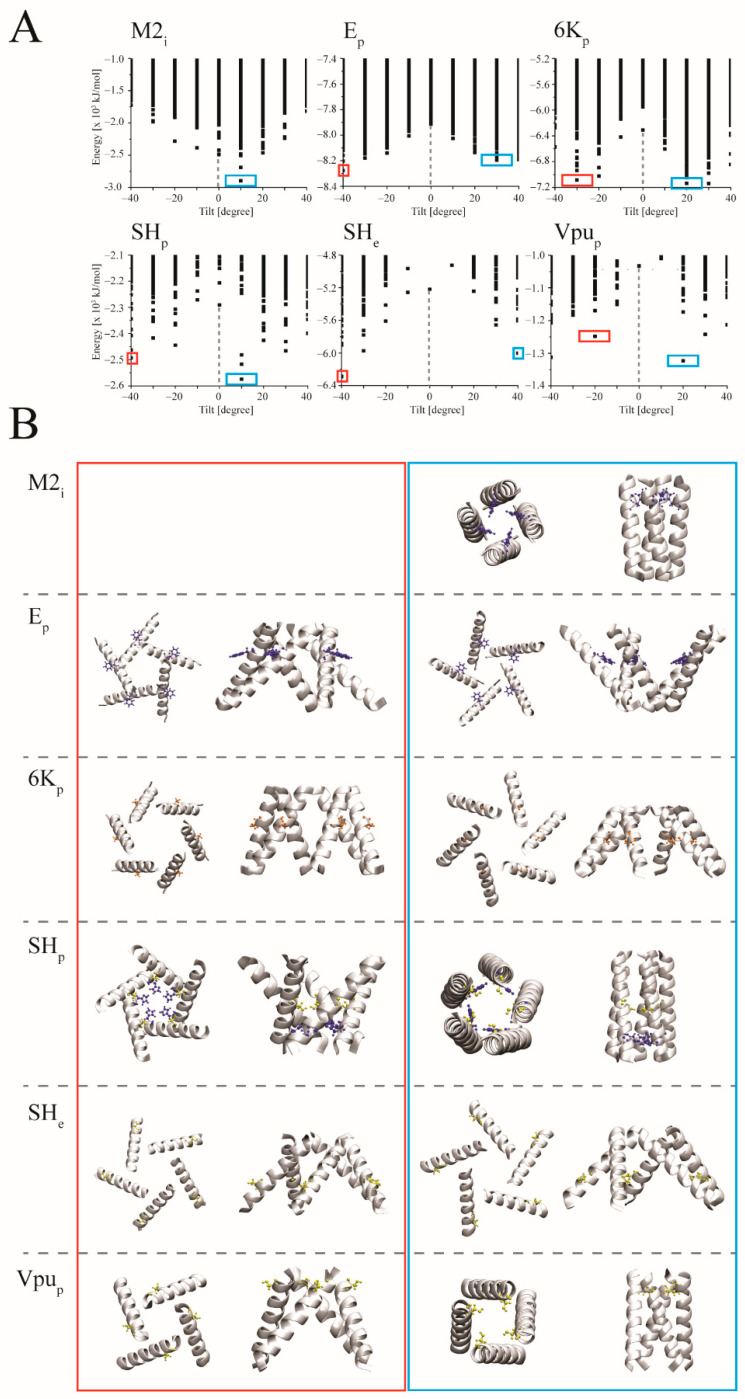
Ranking of the assembled all atom (AA) bundles over the tilt and selected structural models using S1 protocol. (**A**) The estimated binding energies (EBEs) of tetrameric bundles using ideal TMDs of M2_i_ and Vpu_p_, the pentameric bundles of E_p_, SH_p_, SH_e_, and the hexameric bundle of 6K_p_ are plotted over the tilt. The grey dashed line marks the EBE for a zero tilt. The blue and red boxes mark minimum energies for energies with tilts larger and lower, respectively, than tilt values with zero tilt. (**B**) Structural models of the respective bundles marked with the red (tilt < 0 tilt) and blue boxes (tilt > 0) in top (from N to C) and side view (cytoplasmic side pointing downwards). The helix- backbone is shown in grey cartoon mode. Side chains mark the following residues: H37 for M2 and F26 for E, as well as A28 for 6K; H22 and S29 for SH_p_; S29 only for SH_e_; and S24 for Vpu.

A single minimum EBE-structure for a left-handed tetrameric AA-bundle is observed for M2_i_ when applying the S1 protocol (Figure 1A). This first ranked structure (1r) reveals that the functional important residue H37, here mr, is pointing into the pore and that the handedness matches the handedness identified in the experimental structure (Figure 1B and Table 1, red tile). A similar result of correct handedness and orientation with the 1r-structure is obtained when using the TMDs of the crystal structures, M2_e_ (Table 1, red tile). In the case of the E_p_ protein, there are pseudo-minima structures for either handedness of the AA-models with lower EBE values for the right-handed structure (Figure 1A,B). This structure adopts the handedness as shown in the experimental structure. It is the structure ranked 53, which adopts in addition also the right orientation with the mr F26 pointing into the pore (Table 1). Undertaking a fine-grained S2 protocol of both the M2 and E models leads to improved EBEs for all of the assemblies (Supplementary Table S1).

**Figure 2:**
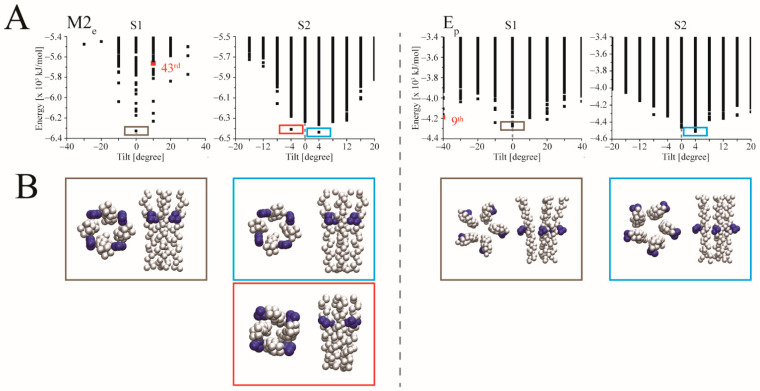
Ranking of coarse-grained (CG) models of tetrameric M2_e_ and pentameric E_p_ plotted over tilt. (**A**) The estimated binding energies (EBEs) of M2_e_ and E_p_ with a minimum value for zero tilt applying S1 protocol (grey box, left side plots for M2_e_ and E_p_) and the subsequent S2 protocol screening around zero tilt (respective right-side plots). Red and blue boxes mark minimum energies for structures with tilts lower and larger, respectively, than tilt values with zero tilt. Energies for structures with the proper orientation of the marker residues are marked as red spheres with the ranking (e.g., 9th) outlined. (**B**) Structural models with the lowest energies in the S1 protocol (grey box) and S2 protocol (red and blue boxes). The marker residues H37 and F26 for M2_e_ and E_p_, respectively, are shown in dark blue spheres.

The S1 protocol for the CG-models of M2_e_ and E_p_, delivers minimum EBEs for the assemblies with TMDs of 0° tilt (Figure 2). A consequent screening using S2, delivers two pseudo-minima tilts with the lowest EBE-structures for both left-handed M2 and E. Whilst the handedness is correct for M2 it is the structure ranked 9th for E_p_ which adopts the proper right-handedness of the bundle. Using AA-MD models does not lead to an improvement of predicting the handedness (Table 1).

The pattern of two pseudo-minima on either side of zero-tilt, is also observed for the assembled TMDs of 6K_p_, SH_p_, SH_e_, and 4Vpu_p_ (Figure 1A). In all these cases the chosen mr’s are identified to point into the pore (Figure 1B). According to the ranking, handedness is proposed to be left-handed for 6K_p_, SH_p_, and 4Vpu_p_, and right-handed for SH_e_ (Supplementary Table S1 and Supplementary Figure S2). In the case of Vpu, assembling the TMDs into tetra-, penta- and hexameric bundles reveals that many of the lowest energy structures of Vpu_28_, Vpu_32_, and Vpu_e_ have S24 pointing into the pore (Table 1 and Supplementary Figure S3). Overwhelmingly, the handedness is found to be right-handed, independent of the model used for the assembly.

Comparison of the docked structures with the experimental structures of M2 and E reveals that RMSD values for left-handed M2 are in the range of 0.42–1.56 nm, and for right-handed E, in the range of 0.45–2.67 nm (Table 1). The tilt angles vary between 6.36°–34.82° for M2 compared to (37.73 ± 0.24)° for M2_e_, and 21.63–53.51° for E proteins compared to (21.69 ± 8.05)° for E_e_.

In comparison with existing software (e.g., Colabfold (CF), GalaxyHomomer (GH) and IntFold, IF) for predicting structure and function of TMDs, PICA provides a tetrameric M2 bundle, M2_e_, as a rank 1 structure closest to the experimental structure in terms of left-handedness (L), RMSD (1.29 nm), position of the mr’s (inside) and tilt (12.20°) (Table 2 and Supplementary Figure S4). Another software, CF, provides a rank 1 structure with data of L/1.34 nm/inside/(30.87 ± 11.76)°, respectively. The rank 1 structure of a third software, GH, shows good RMSD value (0.40 nm) and tilt, as well as correct handedness but the position of the mr’s towards the outside of the bundle. A fourth software, IF, connects all four TMDs into one structure and is therefore not considered further for a comparison of both, M2 and E protein.

In the case of E protein, PICA predicts a right-handed structure as a rank 1 structure, but positions the mr’s towards the outside of the bundle (Table 2 and Supplementary Figure S4). It is the structure ranked 53rd which is the best structure obtained by PICA with values closest to those of the crystal structure. Structures ranked 2nd (CF) and 3rd (GH) predict the proper handedness for the other software but show moderate results for tilt ((1.07 ± 0.36)°, CF) and RMSD (1.56 nm, GH).

The docking protocol unfolds that using ideal structures of the TMDs as in PICA is a reasonable approach to evaluate the handedness of putative bundles. Additionally, CG-models can be used and then transferred back into all atom models for performing S2. Based on the spherical modeling of the amino acids, the CG models allow for more conformational freedom in the ‘almost 2D’ docking approach screening for reliable structures with low EBEs. The decision of which structure to use for S2, is proposed to follow the energy minimum criteria by choosing the overall lowest energy structure 1r. In case of two lower energy structures for either left- or right-handedness both can be carried further applying S2 protocol.

### 3.2. Docking

**Figure 3:**
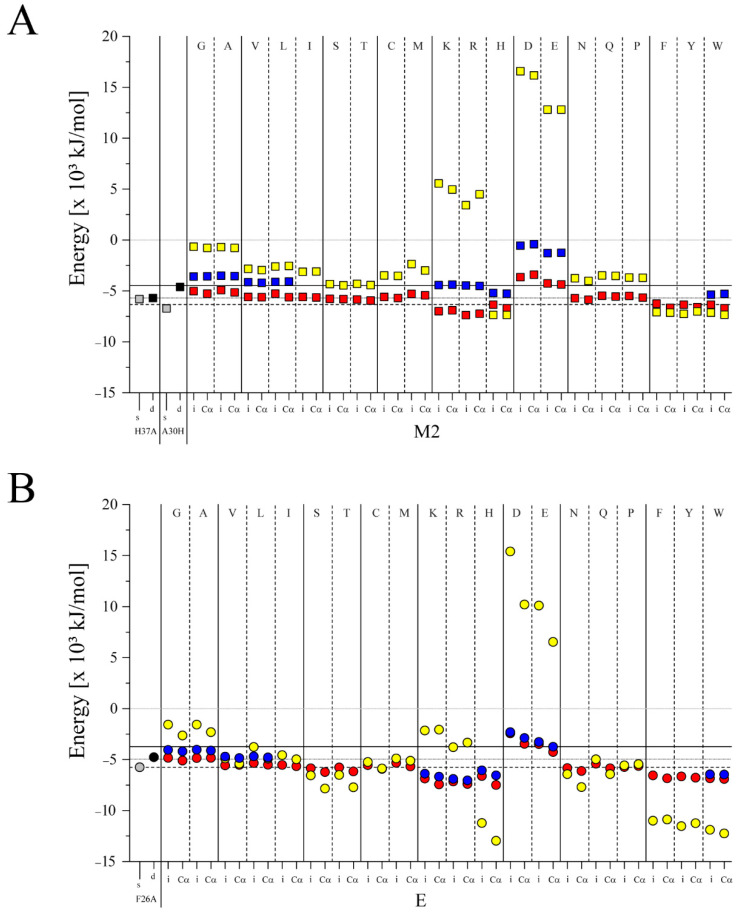
First ranked (1r) estimated binding energies (EBEs) of single helical poly-peptides (pp’s) assembled with target peptides as well as with itself. Each of the single pp’s generated for each of the 20 amino acids is docked to (**A**) M2 (PDB ID: 1NYJ) with the EBE values marked as squares and (**B**) to a truncated version of E (PDB ID: 5X29), E_t_, marking the EBEs with circles. The values are calculated for CG-pp’s modeled either as ideal helices (i) or as helices for which the Cα atoms adopt the same coordinates as the experimental structures (Cα) and being assembled with oligomeric M2 or E bundles. For both plots, individual single pp’s are assembled synchronously (s-screening, s) with three TMDs (1+3) of M2 or four TMDs of E (1+4) (red), as well as 4 (in (**A**)) and 5 pp’s (in (**B**)) with themselves (yellow). In addition, the pp’s are docked using dimeric docking (d-screening, d) in which three or four of the TMDs of M2 and E, respectively, were taken from the respective crystal structures and docked with the pp (blue). Docking of mutants, H37A and A30H for M2 and F26A for E, are shown for s-screening (grey) and d-screening (black). The pp’s are either used as ideal helices, marked with i, or as helices for which the Cα atoms adopt the same coordinates as the experimental structures, marked with Cα. Reference lines are marking the EBEs of (i) the crystal structures of M2_e_ and E_t_ (black lines), (ii) the 1r redocked M2_e_ ad E_t_ structures using s-docking, ^s^M2_e_ and ^s^E_t_, (black dashed line), and (iii) the 1r redocked M2_e_ and E_t_ structures using d-docking, ^d^M2_e_ and ^d^E_t_ (grey line). For EBEs see Supplementary Table S2.

The single pp’s of all 20 amino acids are individually docked with the respective three and four TMDs of M2_e_ and E_t_ protein, respectively, to assess whether EBEs for the 1r structures improve (Figure 3). The rationale is that docking is done with all TMDs and pp’s, either generated as ideal helices, i, or being matched with the coordinates of the backbone Cα-atoms, Cα. The following reference-values are used to evaluate the EBEs: (i) the experimental structures of the proteins in their CG mode ((−4.48 × 10^3^) kJ/mol) for M2, (−3.72 × 10^3^) kJ/mol for E), as well as the redocked experimental structure of the protein either (ii) by following the s-screening protocol (−5.66 × 10^3^) kJ/mol for M2, (−5.60 × 10^3^) kJ/mol for E) or (iii) when docking one of the protein TMDs to the remaining three as described by d-screening for selected amino acids (for M2 (−5.69 × 10^3^) kJ/mol, or four TMDs, for E protein (−4.95 × 10^3^) kJ/mol).

In reference to the EBE of the respective crystal structures and using the s-screening, single mutations of the WT structure lead to improved values for H37A and A30H of M2 (Figure 3A) as well as for F26A of E (Figure 3B). All pp’s independent whether docking in their i- or Cα-mode lead to lower EBEs for both, M2_e_ and E_t_, except for the negatively charged pp’s. In comparison, docking solely the pp’s in this way leads to larger EBEs than the values for the crystal structures independent whether four or five of these peptides are docked.

The differences between 1r-bundles of pp’s docked with the M2 in reference to M2_e_ using the s-screening is on average (e.g., average Δ-value (1.18 ± 0.86) kJ/mol) smaller than the difference between the E’s in reference to E_t_ (averaged Δ-value (1.89 ± 1.14) kJ/mol) (Supplementary Table S2). Additionally, docking with the adopted structure (Cα in Figure 3) improves the EBE values more for the E protein (averaged Δ-value (2.22 ± 1.05) kJ/mol) than for M2 (averaged Δ-value (1.31 ± 0.89) kJ/mol). This pattern is also observed when using the pp’s only.

Calculating the differences in EBEs between the structures in which the mr’s are found to point into the pore, does not alter the pattern described for the 1r structures (Supplementary Figure S5). Calculating averaged EBEs for subgroups of the pp’s, the values are best for subgroups KRH and FYW followed by ST, independent of whether M2 or E bundles are chosen.

**Figure 4:**
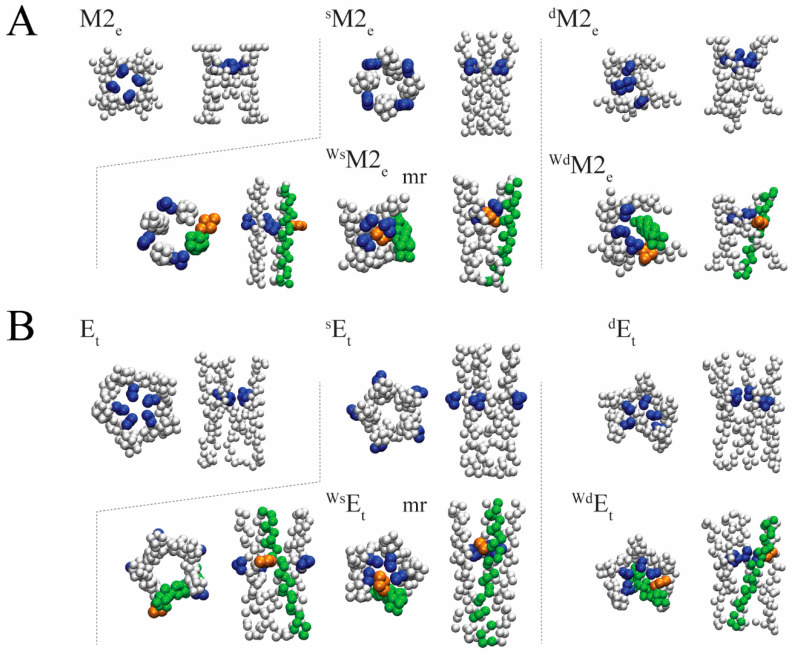
Assembled structures of CG bundles of M2, E TMDs, and with a helical poly-peptide (pp). (**A**) First ranked (1r) tetrameric structures of M2_e_, ^s^M2_e_ and ^d^M2_e_ in a top (left, upper row) and side view (right, upper row) and when assembled with poly-peptide (pp) tryptophan (W), ^Ws^M2_e_ and ^Wd^M2_e_, in its Cα form for which the Cα atoms adopt the same coordinates as the experimental structures (Cα p-peptide) (lower row). (**B**) Pentameric 1r structures of E_t_, redocked structures ^s^E_t_ and ^d^E_t_ (upper row) and as ^Ws^E_t_ and ^Wd^E_t_ (lower row). All bundles are shown in a top view (left side) and side view (right side). “mr” marks the structure with the marker-residues (mr’s) facing the putative pore. Blue spheres mark the mr’s H37 for M2 and F26 for E. The Cα p-peptide with tryptophan, pp-W, is shown in green with one W highlighted in orange to indicate the rotational orientation of the pp-W. All other representative models of the amino acids are shown in grey spheres.

The EBE of the respective crystal structures using d-screening reveals that the selected number of pp’s also improves the EBEs, such as H-pp’s, and W-pp’s for M2 (Supplementary Table S2, data marked with superscript d). The other pp’s do not improve the docking results. In the case of E, all the pp’s show improved results except for the D- and E-pp’s. The docked structures when using the d-screening leads to more compact structures than when using the s-screening (see Figure 4 for using poly-W).

Changing the reference points to those which are obtained by using the redocked reference structures independent of the screening protocol, leaves solely the groups KRH and FYW having improved EBEs (Supplementary Tables S2 and S3, green tiles). The results are indifferent to the type of screening protocol used.

Based on the different docking protocols used and the simplified representation of the ligand by pp’s, it is revealed that KRH and FYW show the best result.

## 4. Discussion

### 4.1. Consideration on the Set-Up of the System

Ideal helices are chosen for the case that no experimental structure is available and they stand for easy-to-access models of TMDs. The use of POPCs as the lipids in the hydrated lipid bilayer system generating the AA-MD models is due to the findings that POPCs are the most abundant lipids in the cellular membranes [71], thus representing a reliable model system. The docking approach is referred to as ‘almost 2D’ docking. This phrase is chosen as much as some degrees such as tilt, tepee-like, pivot point and crossing angle do not ideally representing 2D screening.

PICA handles especially the TMD of membrane proteins and supplements existing programs which predict oligomeric structures on globular proteins (e.g., [72]). It performs very well compared to other server-based programs [68,69,70]. The program is independent of a training set and the necessary disk space only depends on the scale of the screening.

Two types of ff’s are chosen to score the constructed models, one for which all of the aa’s are parameterized, and another one for which the aa’s are represented by simplified spherical models. Since the ff can be freely chosen and implemented into PICA, it can be tuned for novel ff’s, e.g., Martini 3.

### 4.2. Handedness

Docking of the TMDs is conducted by altering the coordinates of all the atoms simultaneously, followed by a steepest descent and conjugated gradient approach. The CG-models are used as a compromise between creating a fast approach and delivering detailed structural information. The CG models allow for reducing the conformational search space after altering the coordinates due to the specification of the CG-model of the TMD and its amino acids. With the CG modeling, the time saving of the docking is in the range of 10 times faster (10% of the time using the fine-grained protocol).

The assembly of the TMDs of M2 and E protein using PICA results in bundles which adopt the same handedness and orientation of the TMDs towards each other like the experimentally derived bundle structures. Most successful is especially the prediction for M2 independent of which model is used. In comparison to E protein, M2 has less residues with bulky side chains such as those of phenylalanine.

Structural information is available for the other TMDs of 6K, SH_p_, SH_e_, and Vpu on the level of a single TMD. Thus, the handedness and relative orientation of the side chains within the bundles are not experimentally resolved. With proposed orientations taken from literature, the assembly delivers especially good results for SH_p_ and SH_e_, as well as for CG-models of Vpu.

In many of the cases, the lowest EBE-structures are not representing the experimental conditions in respect of orientation of the side chains towards the putative center of the bundle. The visually selected structures with a “proper orientation of the side chains” are energetically higher than the best ranked models.

### 4.3. Ligand Docking

The rationale behind the choice of using the 20 pp’s is that at this stage, a screening is done by assessing the efficiency of the individual amino acids in achieving structures with lower EBE than the original bundle structure independent of their sequential alignment in the primary structure of the TMD. The results in this study, that FYW pp’s show improved EBEs, are flanked by the fact that potential drugs contain around two aromatic rings [73].

In the s-screening approach all structures resemble potential pore-like structures in the oligomeric state. In the case of the d-screening, the singled TMD in most cases move closer to the ‘trimer’, with this occluding the putative pore. This indicates that eventually the singled TMD, which would be the potential drug, is leading to an altered assembly eventually not able to full fill the role of the channel.

The improved EBEs for the positively charged pp’s would be especially interesting in as much as a sequence of positively charged amino acids is known to support cell penetration [74,75,76] and are proposed to be relevant as potential drug and cargo translocation facilitators [77]. Using an adequate stretch of these types of amino acids within a putative peptide-based drug would in addition to an improved binding to the membrane also support membrane targeting and insertion-only of these peptides. It is therefore proposed that amino acids in combination with these charged and aromatic residues, will make potential TMD-antivirals.

## 5. Conclusions

The lipid membrane imposes a spatial restriction on the TMDs of membrane proteins, confining the dynamics and orientation of a TMD almost into 2D. Implementing these restrictions, the conformational search-space can be reduced when identifying putative assemblies or bundle structures of TMDs. Reasonable results are obtained when implementing the 2D-like restrictions into a docking software. The docking approach presented enables structure prediction and also enables its application in drug development, especially for membrane-based peptide drugs.

## Figures and Tables

**Table 1:** Ranking of the oligomeric structures based on the estimated binding energy of M2_i_, M2_e_, E_p_, E_e_, 6K_p_, SH_p_, SH_e_, Vpu_28_, Vpu_32_, Vpu_e_ and Vpu_p_. The green-colored tiles indicate that the marker-residues (mr’s) are pointing inside irrespective of the handedness. Numbers indicate the absolute rank according to the structure for which mr’s are within the pore and the handedness of the bundle is correct. The subscripts indicate: e = structures or sequence used from experimental sources; p = predicted sequences that are used to model the transmembrane domain (TMD); 32 and 28 = number of amino acids used for modeling ideal TMDs. The following ideal models are used: AA = all atom models; CG = coarse grained models; AA-MD = all atom models used in 200 ns MD simulation. The “oligomers” are sorted according to the number of TMDs forming the bundle. The “-” indicates that a structure cannot be identified in which the mr’s are pointing into the pore. Numbers in brackets represent the root mean square deviation (RMSD) values in nm of the structures with reference to the experimental structures. Letters R and L refer to right- and left-handed bundles, respectively, of the crystal structures. Numbers in squared brackets represent the tilt angles (°), given as average with standard deviation for M2_e_ and E_e_ proteins. “*” refers to models with the correct handedness and mr pointing into the bundle.

	Oligomer	AA	CG	AA-MD
M2	4_i_	1 * (1.29, L) [12.20]	19 * (1.22, L) [6.36]	4 * (1.66, L) [34.82]
	4_e_ (L) [37.73 ± 0.24]	1 * (0.42, L) [10.04]	43 * (0.64, L) [13.29]	20 * (1.56, L) [22.76]
E	5_p_	53 * (0.45, R) [32.24]	9 * (0.83, R) [53.51]	51 * (1.87, R) [40.68]
	5_e_ (R) [21.69 ± 8.05]	12 * (2.67, R) [24.31]	45 * (2.33, R) [21.63]	-
6K_p_	6	1	59	1
SH_p_	5	1	1	15
SH_e_	5	1	1	1
Vpu_32_	4	7	2	244
	5	3	1	145
	6	3	1	24
Vpu_28_	4	14	1	67
	5	14	1	4
	6	15	2	15
Vpu_e_	4	6	1	41
	5	19	1	2
	6	8	3	1
Vpu_p_	4	1	2	5
	5	2	16	12
	6	2	1	2

**Table 2:** Comparative ranking of the oligomeric structures based on the estimated binding energy of M2 and E protein, M2_e_ and E_e_, in case of PICA, using PICA, ColabFold (CF) and GalaxyHomomer (GH) with experimental structures of M2 (PDB ID: 1NYJ) and E protein (PDB ID: 5X29). For PICA all atom (AA) models of M2_e_ and E_e_ are used using the same amino acid sequence as for M2_i_ and E_p_, respectively. For all other server’s amino acid sequences, M2_i_ and E_p_ are used. The numbers in brackets represent the root mean square deviation (RMSD) values in nm of the structures with reference to the experimental structures. Letters R and L refer to right- and left-handed bundles of the crystal structures. Numbers in squared brackets represent the tilt angles (°), given as average with standard deviation for M2_e_ and E_e_ proteins. “-” = straight TMDs; no handedness. The subscripts indicate the use of: i = ideal helix used; e = sequence as in experimental study reported in literature or experimental structure used for which PDB ID is available; p = ideal structures from sequences of amino acids predicted to form a helical TMD. The best models of PICA compared with the experimental structure are shown in bold.

M2					
Crystal Structure (L) [37.73 ± 0.24]					
	Rank 1	Rank 2	Rank 3	Rank 4	Rank 5
PICA (M2_e_)	**(1.29, L)** **[12.20]**	(1.28, L) [12.11]	(1.30, L) [12.30]	(1.30, -) [3.99]	(1.35, -) [5.19]
CF	(1.34, L) [30.87 ± 11.76]	(1.33, L) [24.32 ± 0.06]	(0.41, L) [30.53 ± 1.68]	(0.34, L) [27.49 ± 1.04]	(1.61, -) [88.83 ± 51.20]
GH	(0.40, L) [28.15 ± 0.30]	(0.32, L) [33.68 ± 13.72]	(0.88, R) [5.52 ± 2.11]	(0.60, L) [26.69 ± 2.89]	(1.11, R) [27.42 ± 3.55]
**E**					
**Crystal structure** **(R)** **[21.69 ± 8.05]**					
PICA (E_e_)	(0.98, R) [48.10]	(1.22, L) [43.51]	(1.26, R) [54.51]	(1.29, L) [47.94]	(1.62, L) [54.16]
	**Rank 53** **(0.45, R)** **[32.24]**				
CF	(1.51, L) [1.07 ± 0.36]	(1.49, R) [3.02 ± 0.48]	(1.38, L) [7.29 ± 0.32]	(1.50, R) [7.48 ± 3.72]	(1.62, R) [23.68 ± 7.25]
GH	(1. 14, L) [30.47 ± 1.44]	(1.11, L) [25.84 ± 5.63]	(1.56, R) [24.27 ± 1.55]	(0.72, R) [11.60 ± 2.53]	(0.62, R) [19.71 ± 0.86]

## Supplementary Materials

The following supporting information can be downloaded at: https://www.mdpi.com/article/10.3390/biom12121844/s1, Supplementary Table S1: Estimated binding energies (EBEs) of the ranked structures of M2, E, 6K, SH, and Vpu; Supplementary Table S2: Estimated binding energies (EBEs) of assembled bundles of TMDs of M2 and E together with each of the poly-peptides; Supplementary Table S3: Averaged estimated binding energies (EBEs) of assembled bundles of TMDs of M2, E and in combination with groups of the poly-peptides; Supplementary Table S4: Estimated binding energy differences (ΔEBEs) for differently assembled poly-peptides (pp’s) and pp-bundles; Supplementary Table S5: Estimated binding energy differences (ΔEBEs) between groups of assembled TMDs; Supplementary Figure S1: Root mean square displacement values over Cα atoms of each structure calculated every 500 ps; Supplementary Figure S2: Ranking of the assembled bundles based on estimated binding energies (EBEs) over tilt from applying S1 protocol and visualization of the structures; Supplementary Figure S3: Ranking of the assembled bundles based on estimated binding energies (EBEs) over tilt from applying S1 protocol and visualization of the structures of Vpu; Supplementary Figure S4: Comparison of assembled tetrameric M2, and pentameric E protein; Supplementary Figure S5: Ranked estimated binding energies (EBEs) of single helical poly-peptides (pp’s) assembled with target peptides as well as with itself.
