# Supplementary material for: Predicting the Assembly of the Transmembrane Domains of Viral Channel Forming Proteins and Peptide Drug Screening Using a Docking Approach
Source: Biomolecules. 2022 Dec 10;12(12):1844. doi: 10.3390/biom12121844 (PMC9775931; doi:10.3390/biom12121844)
Supplement: Supplementary file 1 [file biomolecules-12-01844-s001.zip › biomolecules-2059268-supplementary.pdf]

Predicting the assembly of the transmembrane domains of viral channel forming proteins and peptide drug screening using a docking approach.

Ta-Chou Huang and Wolfgang B. Fischer*

Institute of Biophotonics, School of Biomedical Science and Engineering, National Yang Ming Chiao Tung University, Taipei, Taiwan

*Correspondence to: W. B. Fischer, Institute of Biophotonics, School of Biomedical Science and Engineering, National Yang-Ming University, 155, Li-Nong St., Sec. 2, Taipei, 112, Taiwan

E-mail address: wfischer@nycu.edu.tw

Tel: +886-2-2826-7394, Fax: +886-2-2823-5460

Keywords: assembly structure prediction, viral membrane proteins, transmembrane domain, docking, peptide screening, molecular dynamics simulations.

Table S1: Estimated binding energies (EBEs) of the ranked structures of M2, E, 6K, SH, and Vpu. The energies are sorted according to the structural models being used, all atom (AA), coarse grained (CG) or all atom model after 200 ns of MD simulation (AA-MD). S1 and S2 define the rough and the fine screening, respectively. The bundles are discriminated according to their handedness, right (R), left (L) or straight (0), and whether the respective marker-amino acids are facing into the pore (rank in parenthesis). Bundles for which both, handedness and orientation of the marker-residues, are according to the definition are marked in italics. The number sign '#' marks the first rank structure, but with none of the marker-residues (mr's) facing the pore. The subscripts indicate the use of: e = structures or sequence used from experimental sources; p = ideal structures from sequences of amino acids predicted to form a helical TMD; i = ideal helices; 32 and 28 = numbers of amino acids used for modeling ideal TMDs. The 'oligomers' are sorted according to the number of TMDs forming the bundle.

M2	S1<br>R	0	L	S2<br>R	0	L
AA$_i$			*–2896*			*–3201*
CG$_i$		–3709 (19)	–3992[#]	–4118[#]		–4051[#]
AA-MD$_i$	–4028[#]		*–3891 (4)*	–4251[#]		*–4098*
AA$_e$			*–7471*			*–7912*
CG$_e$		–6327[#]	*–5662 (43)*			–6435[#] (-)
AA-MD$_e$	–8677[#]		*-8284 (20)*	–8919[#]		*–8581*

E	S1<br>R	0	L	S2<br>R	0	L
AA$_p$	–8277[#]<br>*–8083 (53)*			–8428[#] (-)		–8333[#]
CG$_p$	*–4183 (9)*	-4275[#]				–4507[#] (-)
AA-MD$_p$	*–7913 (51)*		–8201	–8294[#]		–8381
AA$_e$	*–25,339 (12)*		–26,338	*–25828*		–26,755
CG$_e$	–13562[#]<br>*–12,293 (45)*			–14081[#]		–13841
AA-MD$_e$			–27,226[#]	–27108[#]		–27,732[#]

Supplementary Table S1 (cont.)

6K	S1<br>R	0	L
AA_p			−7137 (1)
CG_p		−6145 (21)	−6414#
AA-MD_p			−8575 (1)

SH	S1<br>R	0	L
AA_p			−2574 (1)
CG_p			−4226 (1)
AA-MD_p	−4421#		−4070 (15)
AA_e	−6291 (1)		
CG_e			−5225 (1)
AA-MD_e	−6390 (1)		

Supplementary Table S1 (cont.)

Vpu	S1<br>R	0	L	S1<br>R	0	L
	**Vpu$_{28}$ AA**			**Vpu$_{32}$ AA**		
4	−2877$^{\#}$		−2732 (14)	−7579$^{\#}$<br>−7141 (8)		
5	−3411 (14)		−3642$^{\#}$	−9415$^{\#}$<br>−9599 (3)		
6	−4122 (15)	−4289.20$^{\#}$		−11,049 (3)		−11,230.41#

	**Vpu$_{28}$ CG**			**Vpu$_{32}$ CG**		
4			−3100 (1)		−6324$^{\#}$<br>−6286 (2)	
5		−4152 (1)			−8437 (1)	
6		−4859 (2)	−4863$^{\#}$		−10178 (1)	

	**Vpu$_{28}$<br>AA-MD**			**Vpu$_{32}$<br>AA-MD**		
4	−3371$^{\#}$<br>−3187 (67)			−7915$^{\#}$<br>−6950 (244)		
5	−4348$^{\#}$		−4165 (4)	−10,216$^{\#}$		−9161 (145)
6	−5190$^{\#}$		−4930 (15)	−12,381$^{\#}$		−11,483<br>(24)

Supplementary Table S1 (cont.)

Vpu	S1<br>R	0	L	S1<br>R	0	L
	**Vpu_p AA**			**Vpu_e AA**		
4			−1323 (1)	−1109[#]<br>−1023 (6)		
5	−1843[#]		−1832 (2)	−1294 (19)		−1575[#]
6	−2041[#]		−2039 (2)	−1564 (8)		−1772.41[#]

	**Vpu_p AA**			**Vpu_e CG**		
4	−2626[#]		−2593 (2)			−2377 (1)
5	−3249 (16)		−3383[#]			−3122 (1)
6			−4022 (1)	−3633[#]		−3585 (3)

	**Vpu_p<br>AA-MD**			**Vpu_e<br>AA-MD**		
4	−2555.72[#]		−2479 (5)	−2132[#]<br>−2000 (41)		
5	−3440[#]<br>−3178 (5)			−2839[#]		−2704 (2)
6	−3831 (2)		−3,841[#]			−3235 (1)

Supplementary Table S1 (cont.)

Table S2: Estimated binding energies (EBEs) of assembled bundles of TMDs of tetrameric M2 and pentameric E together with each of the poly-peptides (pp's) of all 20 amino acids. The values are calculated for CG-models of pp's as either ideal helices (i, see also in Figure 3) or as helices for which the Cα atoms adopt the same coordinates as the experimental structures (Cα, see also in Figure 3) being assembled with oligomeric M2 or E bundles. The values are calculated in reference to bundles of M2 and E and listed as difference values (ΔEBE) between the docked value and one of the reference values. EBE are calculate for the first ranked bundles (1r) as well for those for which the marker-residues (mr's) are pointing into the putative pore. The ΔEBE are calculates for M2 and E by ($X_{e/t}$ – X_{1r}), ($X_{e/t}$ – X_{mr}), ($^{s}X_{e/1r}$ – X_{1r}), ($^{s}X_{e/1r}$ – X_{mr}), ($^{s}X_{e/mr}$ – X_{1r}), ($^{s}X_{e/mr}$ – X_{mr}), ($^{d}X_{e}$ – X_{1r}), and ($^{d}X_{e}$ – X_{mr}) where X refers to the TMD of the protein, here M2 or E. The letters mark the subscripts used for either M2 and E. The superscripts denote: s and d, for applying synchronous docking (s, s-screening) or dimeric docking (d, d-screening), respectively; the amino acids in single letter code which are used to generate the poly-peptides; /1+3 and /1+4 indicate that synchronous protocol is used to dock a single TMD and a trimeric and tetrameric assembly from the experimental structures of M2 and E, respectively. Subscript '/1r' indicates that the first ranked structure is used and subscript '/mr', that the structure for which the mr's are pointing into the putative pore; t = a truncated version of the protein is used. Colored tiles indicate that the EBE is improved (e.g. $\Delta E_{C\text{-}1r} > 0$) compared to the refence structure (e.g. ΔE_{t}). The colors coding is as follows: orange ≡ reference to $M2_{e}$ / E_{t}; blue ≡ reference to $^{s}M2_{e/1r}$ / $^{s}E_{t/1r}$; yellow ≡ reference to $^{s}M2_{e/mr}$ / $^{s}E_{t/mr}$; green ≡ reference to $^{d}M2_{e}$ / $^{d}E_{t}$.

	Model	1r	mr	ΔEBE							
				$X_{e/t}$ – X_{1r}	$X_{e\ /t}$ – X_{mr}	$^{s}X_{e/1r}$ – X_{1r}	$^{s}Xe_{/1r}$ – X_{mr}	$^{s}X_{e/mr}$ – X_{1r}	$^{s}Xe_{/mr}$ – X_{mr}	$^{d}X_{e}$ – X_{1r}	$^{d}X_{e}$ – X_{mr}
M2	$M2_e$	–4.48	-	-	-	-	-	-	-	-	-
	$^{s}M2_{e/1r}$	–6.33		1.85	-	-	-	-	-	-	-
	$^{s}M2_{e/mr}$	–5.66	-	1.18	-	-	-	-	-	-	-
	$^{d}M2_{e}$	–5.69	-	1.21	-	0.02	-	0.02	-	-	-
M2 mutant	$^{s}H37A_{e}$	–4.68	–4.20	0.20	-0.28	–1.65	–2.13	–0.99	–1.47	–1.01	–1.49
	$^{s/1+3}H37A_{e}$	–5.79	–5.79	1.31	1.31	0.53	–0.53	0.13	0.13	0.10	0.10
	$^{d}H37A_{e}$	–5.70	-	1.22	-	–0.63	-	0.04	-	0.01	-
	$^{s}A30H_{e}$	–5.46	–4.90	0.98	0.42	–0.87	–1.43	–0.20	–0.77	–0.23	–0.79
	$^{s/1+3}A30H_{e}$	–6.72	–6.34	2.24	1.87	0.39	0.02	1.06	0.68	1.03	0.66
	$^{d}A30H_{e}$	–4.61	-	0.13	-	-1.72	-	-1.06	-	-1.08	-
E	E_t	–3.72	-	-	-	-	-	-	-	-	-
	$^{s}E_{t/1r}$	–5.75	-	2.02	-	-	-	-	-	-	-
	$^{s}E_{t/mr}$	–5.60	-	1.88	-	-	-	-	-	-	-
	$^{d}E_{t}$	–4.95	-	1.23	-	–0.65	-	–0.65	-	-	-
E mutant	$^{s}F26A_{t}$	–5.49	–5.22	1.76	1.49	–0.26	–0.53	–0.12	–0.38	0.53	0.27
	$^{s/1+4}F26A_{t}$	–5.77	–5.58	2.04	1.86	0.02	-0.17	0.16	–0.02	0.81	0.63
	$^{d}F26A_{t}$	–4.78	-	1.06	-	–0.96	-	–0.82	-	–0.17	-
Poly-G	$^{Gs}M2_{i}$	–5.01	–4.93	0.54	0.45	–1.31	–1.40	–0.65	–0.74	–0.67	–0.76
	$^{Gs}M2_{C\alpha}$	–5.26	–4.90	0.78	0.42	–1.07	–1.43	–0.41	–0.76	–0.43	–0.79
	$^{Gd}M2_{i}$	–3.59	-	–0.89	-	–2.73	-	–2.07	-	–2.09	-
	$^{Gd}M2_{C\alpha}$	–3.56	-	–0.92	-	–2.77	-	–2.10	-	–2.13	-
	$^{Gs}E_{i}$	–4.84	–4.84	1.12	1.12	–0.90	–0.90	–0.76	–0.76	–0.11	–0.11
	$^{Gs}E_{C\alpha}$	–5.09	–4.93	1.37	1.21	–0.66	–0.82	–0.51	–0.67	0.14	–0.02
	$^{Gd}E_{i}$	–4.05	-	0.33	-	–1.70	-	–1.55	-	–0.90	-
	$^{Gd}E_{C\alpha}$	–4.19	-	0.47	-	–1.56	-	–1.41	-	–0.76	-
Poly-A	$^{As}M2_{i}$	–4.91	–4.79	0.44	0.31	–1.41	–1.54	–0.75	–0.88	–0.77	–0.90
	$^{As}M2_{C\alpha}$	–5.15	–4.93	0.67	0.45	–1.18	–1.40	–0.52	–0.74	–0.54	–0.76
	$^{Ad}M2_{i}$	–3.51	-	–0.97	-	–2.81	-	–2.15	-	–2.17	-
	$^{Ad}M2_{C\alpha}$	–3.54	-	–0.94	-	–2.79	-	–2.13	-	–2.15	-
	$^{As}E_{i}$	–4.85	–4.68	1.13	0.96	–0.89	–1.06	–0.75	–0.92	–0.10	–0.27
	$^{As}E_{C\alpha}$	–4.84	–4.59	1.12	0.86	–0.90	–1.16	–0.76	–1.01	–0.11	–0.36
	$^{Ad}E_{i}$	–4.04	-	0.32	-	–1.71	-	–1.56	-	–0.91	-
	$^{Ad}E_{C\alpha}$	–4.13	-	0.40	-	–1.62	-	–1.48	-	–0.83	-
Poly-V	$^{Vs}M2_{i}$	–5.59	–5.26	1.11	0.78	–0.74	–1.07	–0.07	–0.40	–0.10	–0.43
	$^{Vs}M2_{C\alpha}$	–5.62	–5.40	1.14	0.92	–0.71	–0.93	–0.04	–0.27	–0.07	–0.29
	$^{Vd}M2_{i}$	–4.13	-	-0.35	-	–2.20	-	–1.53	-	–1.56	-
	$^{Vd}M2_{C\alpha}$	–4.20	-	-0.27	-	–2.12	-	–1.46	-	–1.48	-
	$^{Vs}E_{i}$	–5.57	–5.34	1.85	1.61	–0.18	–0.41	–0.03	–0.26	0.62	0.39
	$^{Vs}E_{C\alpha}$	–5.53	–5.47	1.80	1.74	–0.22	–0.28	–0.07	–0.13	0.58	0.52
	$^{Vd}E_{i}$	–4.72	-	0.99	-	–1.03	-	–0.89	-	–0.23	-
	$^{Vd}E_{C\alpha}$	–4.86	-	1.13	-	–0.89	-	–0.75	-	–0.10	-

Supplementary Table S2 (cont.)

	Model	1r	mr	ΔEBE							
				$X_{e/t}$ – X_{1r}	$X_{e/t}$ – X_{mr}	$^{s}X_{e/1r}$ – X_{1r}	$^{s}Xe_{/1r}$ – X_{mr}	$^{s}X_{e/mr}$ – X_{1r}	$^{s}Xe_{/mr}$ – X_{mr}	$^{d}X_{e}$ – X_{1r}	$^{d}X_{e}$ – X_{mr}
Poly-L	$^{Ls}M2_{i}$	–5.26	–5.17	0.79	0.70	–1.06	–1.15	–0.40	–0.49	–0.42	–0.51
	$^{Ls}M2_{C\alpha}$	–5.61	–5.45	1.13	0.98	–0.72	–0.87	–0.05	–0.21	–0.08	–0.23
	$^{Ld}M2_{i}$	–4.11	-	–0.37	-	–2.21	-	–1.55	-	–1.58	-
	$^{Ld}M2_{C\alpha}$	–4.08	-	–0.40	-	–2.25	-	–1.58	-	–1.61	-
	$^{Ls}E_{i}$	–5.34	–5.22	1.61	1.49	–0.41	–0.53	–0.26	–0.39	0.39	0.27
	$^{Ls}E_{C\alpha}$	–5.51	–5.32	1.79	1.60	–0.23	–0.43	–0.09	–0.28	0.56	0.37
	$^{Ld}E_{i}$	–4.70	-	0.97	-	–1.05	-	–0.91	-	–0.25	-
	$^{Ld}E_{C\alpha}$	–4.79	-	1.07	-	–0.96	-	–0.81	-	–0.16	-
Poly-I	$^{Is}M2_{i}$	–5.58	–5.35	1.11	0.87	–0.74	–0.98	–0.08	–0.31	–0.10	–0.34
	$^{Is}M2_{C\alpha}$	–5.64	–5.37	1.16	0.89	–0.68	–0.96	–0.02	–0.30	–0.04	–0.32
	$^{Is}E_{i}$	–5.54	–5.10	1.82	1.38	–0.21	–0.65	–0.06	–0.50	0.59	0.15
	$^{Is}E_{C\alpha}$	–5.65	–5.34	1.92	1.62	–0.10	–0.40	0.04	–0.26	0.69	0.39
Poly-S	$^{Ss}M2_{i}$	–5.78	–5.55	1.30	1.07	–0.55	–0.78	0.11	–0.12	0.09	–0.14
	$^{Ss}M2_{C\alpha}$	–5.79	–5.69	1.32	1.21	–0.53	–0.64	0.13	0.03	0.11	0.00
	$^{Ss}E_{i}$	–5.87	–5.51	2.15	1.79	0.12	–0.24	0.27	–0.09	0.92	0.56
	$^{Ss}E_{C\alpha}$	–6.22	–6.05	2.50	2.33	0.48	0.31	0.62	0.45	1.27	1.10
Poly-T	$^{Ts}M2_{i}$	–5.84	–5.64	1.36	1.16	–0.49	–0.69	0.17	–0.02	0.15	–0.05
	$^{Ts}M2_{C\alpha}$	–5.94	–5.62	1.46	1.14	–0.39	–0.70	0.27	–0.04	0.25	–0.07
	$^{Ts}E_{i}$	–5.78	–5.61	2.06	1.89	0.03	–0.13	0.18	0.01	0.83	0.66
	$^{Ts}E_{C\alpha}$	–6.17	–5.88	2.45	2.16	0.43	0.13	0.57	0.28	1.22	0.93
Poly-C	$^{Cs}M2_{i}$	–5.59	–5.47	1.11	0.99	–0.74	–0.86	–0.08	–0.19	–0.10	–0.22
	$^{Cs}M2_{C\alpha}$	–5.70	–5.38	1.22	0.91	–0.63	–0.94	0.04	–0.28	0.01	–0.30
	$^{Cs}E_{i}$	–5.56	–5.47	1.83	1.74	-0.19	–0.28	–0.05	–0.14	0.60	0.51
	$^{Cs}E_{C\alpha}$	–5.92	–5.49	2.20	1.77	0.17	–0.26	0.32	–0.11	0.97	0.54
Poly-M	$^{Ms}M2_{i}$	–5.29	–5.29	0.81	0.81	–1.04	–1.04	–0.38	–0.38	–0.40	–0.40
	$^{Ms}M2_{C\alpha}$	–5.42	–5.42	0.94	0.94	–0.91	–0.91	–0.24	–0.25	–0.27	–0.27
	$^{Ms}E_{i}$	–5.33	–5.22	1.61	1.50	–0.42	–0.53	–0.27	–0.38	0.38	0.27
	$^{Ms}E_{C\alpha}$	–5.68	–5.57	1.96	1.84	–0.06	–0.18	0.08	–0.04	0.73	0.61
Poly-K	$^{Ks}M2_{i}$	–6.98	–6.48	2.50	2.01	0.66	0.16	1.32	0.82	1.30	0.80
	$^{Ks}M2_{C\alpha}$	–6.90	–6.60	2.43	2.12	0.58	0.27	1.24	0.93	1.22	0.91
	$^{Kd}M2_{i}$	–4.43	-	–0.05	-	–1.90	-	–1.23	-	–1.26	-
	$^{Kd}M2_{C\alpha}$	–4.38	-	-0.10	-	–1.94	-	–1.28	-	–1.30	-
	$^{Ks}E_{i}$	–6.86	–6.68	3.14	2.96	1.11	0.94	1.26	1.08	1.91	1.73
	$^{Ks}E_{C\alpha}$	–7.45	–7.18	3.72	3.46	1.70	1.43	1.85	1.58	2.50	2.23
	$^{Kd}E_{i}$	–6.40	-	2.67	-	0.65	-	0.79	-	1.44	-
	$^{Kd}E_{C\alpha}$	–6.68	-	2.96	-	0.93	-	1.08	-	1.73	-

Supplementary Table S2 (cont.)

	Model	1r	mr	ΔEBE							
				$X_{e/t}$ – X_{1r}	$X_{e/t}$ – X_{mr}	$^{s}X_{e/1r}$ – X_{1r}	$^{s}Xe_{/1r}$ – X_{mr}	$^{s}X_{e/mr}$ – X_{1r}	$^{s}Xe_{/mr}$ – X_{mr}	$^{d}X_{e}$ – X_{1r}	$^{d}X_{e}$ – X_{mr}
Poly-R	$^{Rs}M2_{i}$	–7.37	–6.54	2.89	2.06	1.04	0.21	1.71	0.88	1.68	0.85
	$^{Rs}M2_{C\alpha}$	–7.24	–6.72	2.76	2.24	0.91	0.39	1.57	1.06	1.55	1.03
	$^{Rd}M2_{i}$	–4.47	-	-0.01	-	-1.86	-	–1.19	-	–1.22	-
	$^{Rd}M2_{C\alpha}$	–4.51	-	0.03	-	-1.82	-	–1.15	-	–1.18	-
	$^{Rs}E_{i}$	–7.15	–6.95	3.43	3.23	1.41	1.21	1.55	1.35	2.20	2.00
	$^{Rs}E_{C\alpha}$	–7.39	–7.39	3.67	3.67	1.64	1.64	1.79	1.79	2.44	2.44
	$^{Rd}E_{i}$	–6.91	-	3.19	-	1.16	-	1.31	-	1.96	-
	$^{Rd}E_{C\alpha}$	–7.05	-	3.33	-	1.31	-	1.45	-	2.10	-
Poly-H	$^{Hs}M2_{i}$	–6.36	–6.36	1.88	1.88	0.03	0.03	0.70	0.70	0.67	0.67
	$^{Hs}M2_{C\alpha}$	–6.71	–6.66	2.23	2.18	0.38	0.33	1.05	1.00	1.02	0.97
	$^{Hd}M2_{i}$	–5.21	-	0.73	-	–1.12	-	–0.45	-	–0.48	-
	$^{Hd}M2_{C\alpha}$	–5.26	-	0.78	-	–1.07	-	–0.40	-	–0.43	-
	$^{Hs}E_{i}$	–6.61	–6.44	2.89	2.71	0.86	0.69	1.01	0.84	1.66	1.49
	$^{Hs}E_{C\alpha}$	–7.51	–7.03	3.78	3.30	1.76	1.28	1.90	1.42	2.56	2.07
	$^{Hd}E_{i}$	–6.06	-	2.33	-	0.31	-	0.45	-	1.10	-
	$^{Hd}E_{C\alpha}$	–6.55	-	2.83	-	0.80	-	0.95	-	1.60	-
Poly-D	$^{Ds}M2_{i}$	–3.64	–3.64	–0.83	–0.83	–2.68	–2.68	–2.02	–2.02	–2.04	–2.04
	$^{Ds}M2_{C\alpha}$	–3.39	–3.39	–1.08	–1.09	–2.93	–2.94	–2.27	–2.28	–2.29	–2.30
	$^{Dd}M2_{i}$	–0.55	-	–3.93	-	–5.78	-	–5.11	-	–5.14	-
	$^{Dd}M2_{C\alpha}$	–0.42	-	–4.06	-	–5.91	-	–5.25	-	–5.27	-
	$^{Ds}E_{i}$	–2.44	–2.44	–1.28	–1.28	–3.31	–3.31	–3.16	–3.16	–2.51	–2.51
	$^{Ds}E_{C\alpha}$	–3.46	–3.28	–0.27	–0.45	–2.29	–2.47	–2.15	–2.33	–1.49	–1.68
	$^{Dd}E_{i}$	–2.32	-	–1.40	-	–3.43	-	–3.28	-	–2.63	-
	$^{Dd}E_{C\alpha}$	–2.90	-	–0.83	-	–2.85	-	–2.71	-	–2.05	-
Poly-E	$^{Es}M2_{i}$	–4.26	–4.26	–0.22	–0.22	–2.06	–2.06	–1.40	–1.40	–1.42	–1.42
	$^{Es}M2_{C\alpha}$	–4.37	–4.37	–0.11	–0.11	–1.96	–1.96	–1.30	–1.30	–1.32	–1.32
	$^{Ed}M2_{i}$	–1.28	-	–3.19	-	–5.04	-	–4.38	-	–4.40	-
	$^{Ed}M2_{C\alpha}$	–1.25	-	–3.23	-	–5.08	-	–4.41	-	–4.44	-
	$^{Es}E_{i}$	–3.48	–3.48	–0.24	–0.24	–2.26	–2.26	–2.12	–2.12	–1.47	–1.47
	$^{Es}E_{C\alpha}$	–4.26	–4.14	0.54	0.41	–1.49	–1.61	–1.34	–1.46	–0.69	–0.81
	$^{Ed}E_{i}$	–3.28	-	–0.44	-	–2.46	-	–2.32	-	–1.67	-
	$^{Ed}E_{C\alpha}$	–3.76	-	0.03	-	–1.99	-	–1.85	-	–1.20	-
Poly-N	$^{Ns}M2_{i}$	–5.71	–5.51	1.23	1.03	–0.62	–0.82	0.05	–0.15	0.02	–0.18
	$^{Ns}M2_{C\alpha}$	–5.87	–5.68	1.39	1.20	–0.46	–0.65	0.21	0.02	0.18	–0.01
	$^{Ns}E_{i}$	–5.84	–5.61	2.11	1.89	0.09	–0.14	0.23	0.01	0.88	0.66
	$^{Ns}E_{C\alpha}$	–6.13	–5.90	2.41	2.17	0.38	0.15	0.53	0.29	1.18	0.94
Poly-Q	$^{Qs}M2_{i}$	–5.46	–5.36	0.99	0.88	–0.86	–0.97	–0.20	–0.30	–0.22	–0.33
	$^{Qs}M2_{C\alpha}$	–5.56	–5.53	1.08	1.05	–0.77	–0.80	–0.10	–0.14	–0.13	–0.16
	$^{Qs}E_{i}$	–5.41	–5.39	1.69	1.67	–0.34	–0.35	–0.19	–0.21	0.46	0.44
	$^{Qs}E_{C\alpha}$	–5.85	–5.85	2.13	2.13	0.10	0.10	0.25	0.25	0.90	0.90

Supplementary Table S2 (cont.)

	Model	1r	mr	ΔEBE							
				$X_{e/t} - X_{1r}$	$X_{e/t} - X_{mr}$	${}^{s}X_{e/1r} - X_{1r}$	${}^{s}Xe_{/1r} - X_{mr}$	${}^{s}X_{e/mr} - X_{1r}$	${}^{s}Xe_{/mr} - X_{mr}$	${}^{d}X_{e} - X_{1r}$	${}^{d}X_{e} - X_{mr}$
Poly-P	${}^{Ps}M2_{i}$	–5.50	–5.43	1.02	0.95	–0.83	–0.90	–0.17	–0.23	–0.19	–0.26
	${}^{Ps}M2_{C\alpha}$	–5.65	–5.65	1.17	1.17	–0.68	–0.68	–0.02	–0.02	–0.04	–0.04
	${}^{Ps}E_{i}$	–5.75	–5.37	2.02	1.65	0.00	–0.38	0.15	–0.23	0.80	0.42
	${}^{Ps}E_{C\alpha}$	–5.64	–5.52	1.91	1.80	–0.11	–0.23	0.04	–0.08	0.69	0.57
Poly-F	${}^{Fs}M2_{i}$	–6.25	–6.10	1.77	1.62	–0.08	–0.23	0.59	0.44	0.56	0.41
	${}^{Fs}M2_{C\alpha}$	–6.66	–6.21	2.18	1.73	0.33	–0.12	1.00	0.55	0.97	0.52
	${}^{Fs}E_{i}$	–6.56	–6.17	2.83	2.44	0.81	0.42	0.95	0.56	1.61	1.21
	${}^{Fs}E_{C\alpha}$	–6.83	–6.67	3.11	2.95	1.09	0.92	1.23	1.07	1.88	1.72
Poly-Y	${}^{Ys}M2_{i}$	–6.36	–6.12	1.88	1.64	0.03	–0.21	0.70	0.46	0.67	0.43
	${}^{Ys}M2_{C\alpha}$	–6.60	–6.36	2.12	1.88	0.28	0.03	0.94	0.70	0.91	0.67
	${}^{Ys}E_{i}$	–6.66	–6.15	2.93	2.43	0.91	0.40	1.05	0.55	1.70	1.20
	${}^{Ys}E_{C\alpha}$	–6.79	–6.63	3.07	2.91	1.04	0.88	1.19	1.03	1.84	1.68
Poly-W	${}^{Ws}M2_{i}$	–6.36	–6.27	1.88	1.79	0.03	–0.06	0.70	0.61	0.67	0.58
	${}^{Ws}M2_{C\alpha}$	–6.70	–6.35	2.22	1.87	0.37	0.02	1.03	0.68	1.01	0.66
	${}^{Wd}M2_{i}$	–5.38	-	0.90	-	–0.95	-	–0.28	-	–0.31	-
	${}^{Wd}M2_{C\alpha}$	–5.29	-	0.81	-	–1.04	-	–0.38	-	–0.40	-
	${}^{Ws}E_{i}$	–6.85	–6.53	3.13	2.80	1.10	0.78	1.25	0.92	1.90	1.58
	${}^{Ws}E_{C\alpha}$	–6.91	–6.75	3.18	3.03	1.16	1.00	1.30	1.15	1.95	1.80
	${}^{Wd}E_{i}$	–6.45	-	2.72	-	0.70	-	0.85	-	1.50	-
	${}^{Wd}E_{C\alpha}$	–6.48	-	2.76	-	0.73	-	0.88	-	1.53	-
All	${}^{all\text{-}s}M2_{i}$	–5.66 ± 0.86	–5.48 ± 0.73	1.18 ± 0.86	1.00 ± 0.73	–0.67 ± 0.86	–0.85 ± 0.73	–0.01 ± 0.86	–0.19 ± 0.73	–0.03 ± 0.86	–0.21 ± 0.73
	${}^{all\text{-}s}M2_{C\alpha}$	–5.79 ± 0.89	–5.58 ± 0.81	1.31 ± 0.89	1.10 ± 0.81	0.54 ± 0.89	–0.74 ± 0.81	0.13 ± 0.89	–0.08 ± 0.81	0.10 ± 0.89	–0.11 ± 0.81
	${}^{all\text{-}d}M2_{i}$	–3.67 ±1.58	-	–0.81 ±1.58	-	-	-	–2.00 ±1.58	-	–2.02 ±1.58	-
	${}^{all\text{-}d}M2_{C\alpha}$	–3.65 ±1.61	-	–0.83 ±1.61	-	-	-	–2.01 ±1.61	-	–2.04 ±1.61	-
	${}^{all\text{-}s}E_{i}$	–5.61 ±1.14	–5.41 ±1.05	1.89 ±1.14	1.69 ±1.05	–0.13 ±1.14	–0.34 ±1.05	0.01 ±1.14	–0.19 ±1.05	0.66 ±1.14	0.46 ±1.05
	${}^{all\text{-}s}E_{C\alpha}$	–5.94 ±1.05	–5.75 ±1.04	2.22 ±1.05	2.03 ±1.04	0.19 ±1.05	0.00 ±1.04	0.34 ±1.05	0.15 ±1.04	0.99 ±1.05	0.80 ±1.04
	${}^{all\text{-}d}E_{i}$	–4.89 ±1.52	-	1.17 ±1.52	-	-	-	–0.71 ±1.52	-	–0.06 ±1.52	-
	${}^{all\text{-}d}E_{C\alpha}$	–5.14 ±1.45	-	1.41 ±1.45	-	-	-	–0.46 ±1.45	-	0.19 ±1.45	-

Supplementary Table S2 (cont.)

Table S3: Averaged estimated binding energies (EBEs) of assembled bundles of TMDs of tetrameric M2, pentameric E and in combination with groups of the polypeptides (pp's). Details of the calculations and the notations are given in Supplementary Table S2.

	Model	1r	mr	ΔEBA							
				$X_{e/t} - X_{1r}$	$X_{e/t} - X_{mr}$	$^{s}X_{e/1r} - X_{1r}$	$^{s}X_{e/1r} - X_{mr}$	$^{s}X_{e/mr} - X_{1r}$	$^{s}X_{e/mr} - X_{mr}$	$^{d}X_{e} - X_{1r}$	$^{d}X_{e} - X_{mr}$
**M2**	$M2_{C}$	−4.48	-	-	-	-	-	-	-	-	-
	$^{s}M2_{E/1r}$	−6.33	-	1.85	-	-	-	-	-	-	-
	$^{s}M2_{E/mr}$	−5.66	-	1.18	-	-	-	-	-	-	-
	$^{d}M2_{E}$	−5.69	-	1.21	-	−0.64	-	0.02	-	-	-
**E_{E}**	$E_{C\text{-}t}$	−3.72	-	-	-	-	-	-	-	-	-
	$^{s}E_{E/1r}$	−5.75	-	2.02	-	-	-	-	-	-	-
	$^{s}E_{E/mr}$	−5.60	-	1.88	-	-	-	-	-	-	-
	$^{d}E_{E}$	−4.95	-	1.23	-	−0.80	-	−0.65	-	-	-
**GA**	$^{GAs}M2_{i}$	−4.96 ± 0.07	−4.86 ± 0.10	0.49 ± 0.07	0.38 ± 0.10	−1.36 ± 0.07	−1.47 ± 0.10	−0.70 ± 0.07	−0.81 ± 0.10	−0.72 ± 0.07	−0.83 ± 0.10
	$^{GAs}M2_{C\alpha}$	−5.20 ± 0.08	−4.91 ± 0.02	0.72 ± 0.08	0.43 ± 0.02	−1.13 ± 0.08	−1.41 ± 0.02	−0.46 ± 0.08	−0.75 ± 0.02	−0.49 ± 0.08	−0.77 ± 0.02
	$^{GAd}M2_{i}$	−3.55 ± 0.06	-	−0.93 ± 0.06	-	−2.77 ± 0.06	-	−2.11 ± 0.06	-	−2.13 ± 0.06	-
	$^{GAd}M2_{C\alpha}$	−3.55 ± 0.02	-	−0.93 ± 0.02	-	−2.78 ± 0.02	-	−2.11 ± 0.02	-	−2.14 ± 0.02	-
	$^{GAs}E_{i}$	−4.85 ± 0.01	−4.76 ± 0.11	1.13 ± 0.01	1.04 ± 0.11	−0.90 ± 0.01	−0.98 ± 0.11	−0.75 ± 0.01	−0.84 ± 0.11	−0.10 ± 0.01	−0.19 ± 0.11
	$^{GAs}E_{C\alpha}$	−4.97 ± 0.18	−4.76 ± 0.24	1.24 ± 0.18	1.04 ± 0.24	−0.78 ± 0.18	−0.99 ± 0.24	−0.63 ± 0.18	−0.84 ± 0.24	0.02 ± 0.18	−0.19 ± 0.24
	$^{GAd}E_{i}$	−4.0 5± 0.01		0.32 ± 0.01	-	−1.70 ± 0.01	-	−1.56 ± 0.01	-	−0.91 ± 0.01	-
	$^{GAd}E_{C\alpha}$	−4.16 ± 0.05		0.44 ± 0.05	-	−1.59 ± 0.05	-	−1.44 ± 0.05	-	−0.79 ± 0.05	-
**VLI**	$^{VLIs}M2_{i}$	−5.48 ± 0.19	−5.26 ± 0.09	1.00 ± 0.19	0.78 ± 0.09	−0.85 ± 0.19	−1.07 ± 0.09	−0.18 ± 0.19	−0.40 ± 0.09	−0.21 ± 0.19	−0.43 ± 0.09
	$^{VLIs}M2_{C\alpha}$	−5.62 ± 0.02	−5.41 ± 0.04	1.15 ± 0.02	0.93 ± 0.04	−0.70 ± 0.02	−0.92 ± 0.04	−0.04 ± 0.02	−0.26 ± 0.04	−0.06 ± 0.02	−0.28 ± 0.04
	$^{VLId}M2_{i}$	−4.12 ± 0.01	-	−0.36 ± 0.01	-	−2.21 ± 0.01	-	−1.54 ± 0.01	-	−1.57 ± 0.01	-
	$^{VLId}M2_{C\alpha}$	−4.14 ± 0.09	-	−0.34 ± 0.09	-	−2.18 ± 0.09	-	−1.52 ± 0.09	-	−1.55 ± 0.09	-
	$^{VLIs}E_{i}$	−5.48 ± 0.13	−5.22 ± 0.12	1.76 ± 0.13	1.50 ± 0.12	−0.27 ± 0.13	−0.53 ± 0.12	−0.12 ± 0.13	−0.38 ± 0.12	0.53 ± 0.13	0.27 ± 0.12
	$^{VLIs}E_{C\alpha}$	−5.56 ± 0.07	−5.38 ± 0.08	1.84 ± 0.07	1.65 ± 0.08	−0.19 ± 0.07	−0.37 ± 0.08	−0.04 ± 0.07	−0.23 ± 0.08	0.61 ± 0.07	0.43 ± 0.08
	$^{VLId}E_{i}$	−4.71 ± 0.01	-	0.98 ± 0.01	-		-	−0.90 ± 0.01	-	−0.24 ± 0.01	-
	$^{VLId}E_{C\alpha}$	−4.82 ± 0.05	-	1.10 ± 0.05	-		-	−0.78 ± 0.05	-	−0.13 ± 0.05	-
**ST**	$^{STs}M2_{i}$	−5.81 ± 0.04	−5.59 ± 0.07	1.33 ± 0.04	1.12 ± 0.07	−0.52 ± 0.04	−0.73 ± 0.07	0.14 ± 0.04	−0.07 ± 0.07	0.12 ± 0.04	−0.09 ± 0.07
	$^{STs}M2_{C\alpha}$	−5.87 ± 0.10	−5.66 ± 0.05	1.39 ± 0.10	1.18 ± 0.05	−0.46 ± 0.10	−0.67 ± 0.05	0.20 ± 0.10	−0.01 ± 0.05	0.18 ± 0.10	−0.03 ± 0.05
	$^{STs}E_{i}$	−5.82 ± 0.06	−5.56 ± 0.07	2.10 ± 0.06	1.84 ± 0.07	0.08 ± 0.06	−0.19 ± 0.07	0.22 ± 0.06	−0.04 ± 0.07	0.87 ± 0.06	0.61 ± 0.07
	$^{STs}E_{C\alpha}$	−6.20 ± 0.04	−5.97 ± 0.12	2.47 ± 0.04	2.24 ± 0.12	0.45 ± 0.04	0.22± 0.12	0.60 ± 0.04	0.37 ± 0.12	1.25 ± 0.04	1.02 ± 0.12

Supplementary Table S3 (cont.)

	Model	1r	mr	ΔEBA							
				$X_{e/t} - X_{1r}$	$X_{e/t} - X_{mr}$	$^{s}X_{e/1r} - X_{1r}$	$^{s}Xe_{/1r} - X_{mr}$	$^{s}X_{e/mr} - X_{1r}$	$^{s}Xe_{/mr} - X_{mr}$	$^{d}X_{e} - X_{1r}$	$^{d}X_{e} - X_{mr}$
**CM**	$^{CMs}M2_{i}$	−5.44 ± 0.21	−5.38 ± 0.13	0.96 ± 0.21	0.90 ± 0.13	−0.89 ± 0.21	−0.95 ± 0.13	−0.23 ± 0.21	−0.29 ± 0.13	−0.25± 0.21	−0.31 ± 0.13
	$^{CMs}M2_{C\alpha}$	−5.56 ± 0.20	−5.40 ± 0.02	1.08 ± 0.20	0.92 ± 0.02	−0.77 ± 0.20	−0.93 ± 0.02	−0.10 ± 0.20	−0.26 ± 0.02	−0.13± 0.20	−0.29 ± 0.02
	$^{CMs}E_{i}$	−5.44 ± 0.16	−5.34 ± 0.17	1.72 ± 0.16	1.62 ± 0.17	−0.30 ± 0.16	−0.4 1± 0.17	−0.16 ± 0.16	−0.26 ± 0.17	0.49 ± 0.16	0.39 ± 0.17
	$^{CMs}E_{C\alpha}$	−5.8 ± 0.17	−5.53 ± 0.05	2.08 ± 0.17	1.80 ± 0.05	0.05 ± 0.17	−0.22 ± 0.05	0.20 ± 0.17	−0.07 ± 0.05	0.85 ± 0.17	0.58 ± 0.05
**KRH**	$^{KRHs}M2_{i}$	−6.91 ± 0.51	−6.46 ± 0.09	2.43 ± 0.51	1.98 ± 0.09	0.58 ± 0.51	0.14 ± 0.09	1.24 ± 0.51	0.80 ± 0.09	1.22 ± 0.51	0.7 7± 0.09
	$^{KRHs}M2_{C\alpha}$	−6.95 ± 0.27	−6.66 ± 0.06	2.47 ± 0.27	2.18 ± 0.06	0.62 ± 0.27	0.33 ± 0.06	1.29 ± 0.27	1.00 ± 0.06	1.26 ± 0.27	0.97 ± 0.06
	$^{KRHd}M2_{i}$	−4.70 ± 0.44	-	0.22 ± 0.44	-	−1.62 ± 0.44	-	−0.96 ± 0.44	-	−0.99 ± 0.44	-
	$^{KRHd}M2_{C\alpha}$	−4.72 ± 0.47	-	0.24 ± 0.47	-	−1.61 ± 0.47	-	−0.95 ± 0.47	-	−0.97 ± 0.47	-
	$^{KRHs}E_{i}$	−6.88 ± 0.27	−6.69 ± 0.26	3.15 ± 0.27	2.97 ± 0.26	1.13 ± 0.27	0.94 ± 0.26	1.27 ± 0.27	1.09 ± 0.26	1.92± 0.27	1.74 ± 0.26
	$^{KRHs}E_{C\alpha}$	−7.45 ± 0.06	−7.20 ± 0.18	3.73 ± 0.06	3.47 ± 0.18	1.70 ± 0.06	1.45 ± 0.18	1.85 ± 0.06	1.60 ± 0.18	2.50 ± 0.06	2.25 ± 0.18
	$^{KRHd}E_{i}$	−6.45 ± 0.43	-	2.73 ± 0.43	-	0.71 ± 0.43	-	0.85 ± 0.43	-	1.50 ± 0.43	-
	$^{KRHd}E_{C\alpha}$	−6.76 ± 0.26	-	3.04 ± 0.26	-	1.01 ± 0.26	-	1.16 ± 0.26	-	1.81 ± 0.26	-
**DE**	$^{DEs}M2_{i}$	−3.95 ± 0.44	−3.95 ± 0.44	−0.52 ± 0.44	−0.52 ± 0.44	−2.37 ± 0.44	−2.37 ± 0.44	−1.71 ± 0.44	−1.71 ± 0.44	−1.73 ± 0.44	−1.73 ± 0.44
	$^{DEs}M2_{C\alpha}$	−3.88 ± 0.69	−3.88 ± 0.69	−0.60 ± 0.69	−0.60 ± 0.69	−2.45 ± 0.69	−2.45 ± 0.69	−1.78 ± 0.69	−1.79 ± 0.69	−1.81 ± 0.69	−1.81 ± 0.69
	$^{DEd}M2_{i}$	−0.92 ± 0.52	-	−3.56 ± 0.52	-	−5.41 ± 0.52	-	−4.75 ± 0.52	-	−4.77 ± 0.52	-
	$^{DEd}M2_{C\alpha}$	−0.83 ± 0.59	-	−3.65 ± 0.59	-	−5.50 ± 0.59	-	−4.83 ± 0.59	-	−4.86 ± 0.59	-
	$^{DEs}E_{i}$	−2.96 ± 0.74	−2.96 ± 0.74	−0.76 ± 0.74	−0.76 ± 0.74	−2.79 ± 0.74	−2.79 ± 0.74	−2.64 ± 0.74	−2.64 ± 0.74	−1.99 ± 0.74	−1.99 ± 0.74
	$^{DEs}E_{C\alpha}$	−3.86 ± 0.57	−3.71 ± 0.61	0.14 ± 0.57	−0.02 ± 0.61	−1.89 ± 0.57	−2.04 ± 0.61	−1.74 ± 0.57	−1.89 ± 0.61	−1.09± 0.57	−1.24 ± 0.61
	$^{DEd}E_{i}$	−2.80 ± 0.68	-	−0.92 ± 0.68	-	−2.95 ± 0.68	-	−2.80 ± 0.68	-	−2.15 ± 0.68	-
	$^{DEd}E_{C\alpha}$	−3.33 ± 0.61	-	−0.40 ± 0.61	-	−2.42 ± 0.61	-	−2.28 ± 0.61	-	−1.63 ± 0.61	-
**NQP**	$^{NQPs}M2_{i}$	−5.56 ± 0.13	−5.43 ± 0.08	1.08 ± 0.13	0.95 ± 0.08	−0.77 ± 0.13	−0.89 ± 0.08	−0.11 ± 0.13	−0.23 ± 0.08	−0.13 ± 0.13	−0.26± 0.08
	$^{NQPs}M2_{C\alpha}$	−5.69 ± 0.16	−5.62 ± 0.08	1.21 ± 0.16	1.14 ± 0.08	−0.64 ± 0.16	−0.71 ± 0.08	0.03 ± 0.16	−0.05 ± 0.08	0.00 ± 0.16	−0.07± 0.08
	$^{NQPs}E_{i}$	−5.67 ± 0.22	−5.46 ± 0.13	1.94 ± 0.22	1.73 ± 0.13	−0.08 ± 0.22	−0.29 ± 0.13	0.06 ± 0.22	−0.14 ± 0.13	0.71 ± 0.22	0.51 ± 0.13
	$^{NQPs}E_{C\alpha}$	−5.87 ± 0.25	−5.76 ± 0.20	2.15 ± 0.25	2.03 ± 0.20	0.13 ± 0.25	0.01 ± 0.20	0.27 ± 0.25	0.15 ± 0.20	0.92 ± 0.25	0.81 ± 0.20

Supplementary Table S3 (cont.)

	Model	1r	mr	ΔEBA							
				$X_{e/t} - X_{1r}$	$X_{e/t} - X_{mr}$	${}^{s}X_{e/1r} - X_{1r}$	${}^{s}X_{e/1r} - X_{mr}$	${}^{s}X_{e/mr} - X_{1r}$	${}^{s}X_{e/mr} - X_{mr}$	${}^{d}X_{e} - X_{1r}$	${}^{d}X_{e} - X_{mr}$
**F Y W**	${}^{FYWs}M2_{i}$	−6.32 ± 0.06	−6.16 ± 0.09	1.84 ± 0.06	1.68 ± 0.09	0.00 ± 0.06	−0.16 ± 0.09	0.66 ± 0.06	0.50 ± 0.09	0.64 ± 0.06	0.48 ± 0.09
	${}^{FYWs}M2_{C\alpha}$	−6.65 ± 0.05	−6.31 ± 0.08	2.17 ± 0.05	1.83 ± 0.08	0.33 ± 0.05	−0.02 ± 0.08	0.99 ± 0.05	0.64 ± 0.08	0.97 ± 0.05	0.62 ± 0.08
	${}^{FYWd}M2_{i}$	−5.38	-	0.90	-	−0.95	-	−0.28	-	−0.31	-
	${}^{FYWd}M2_{C\alpha}$	−5.29	-	0.81	-	−1.04	-	−0.38	-	−0.40	-
	${}^{FYWs}E_{i}$	−6.69 ± 0.15	−6.28 ± 0.21	2.96 ± 0.15	2.56 ± 0.21	0.94 ± 0.15	0.53 ± 0.21	1.09 ± 0.15	0.68 ± 0.21	1.74 ± 0.15	1.33 ± 0.21
	${}^{FYWs}E_{C\alpha}$	−6.84 ± 0.06	−6.68 ± 0.06	3.12 ± 0.06	2.96 ± 0.06	1.10 ± 0.06	0.93 ± 0.06	1.24 ± 0.06	1.08 ± 0.06	1.89 ± 0.06	1.73 ± 0.06
	${}^{FYWd}E_{i}$	−6.45	-	2.72	-	0.70	-	0.85	-	1.50	-
	${}^{FYWd}E_{C\alpha}$	−6.48	-	2.76	-	0.73	-	0.88	-	1.53	-

Supplementary Table S3 (cont.)

Table S4: Estimated binding energy differences (ΔEBEs) for differently assembled poly-peptides (pp's) and pp-bundles. The difference is calculated between (i) the EBEs of CG models of bundles in which the to-be-docked pp and pp-bundles are modeled as ideal helix (i, see also in Figure 3) and (ii) the EBEs when modeled with TMDs and pp's for which the Cα atoms are adopted to the same coordinates as the experimental structures (Cα, see also in Figure 3). '1r' and 'mr' mark the values taken from first ranked structures of trimeric M2 and tetrameric E experimental bundles M2$_e$ and E$_t$ and those for which the marker-residues are pointing into the putative pore, respectively. The trimeric and tetrameric bundles are obtained by deleting one helix from the bundle using the software MOE. 'aa' marks the amino acid the pp is made of. (in superscript as aa/) and whether synchronous docking (s, s-screening) or dimeric docking (d, d-screening) is applied, shown as e.g. 'aa/s'. e = structures or sequence used from experimental sources; t = a truncated version of E protein is used; 4 and 5 mark whether a tetrameric ensemble or pentameric ensemble is modeled. The number of TMDs used for the poly-peptides are marked as superscripts 4/ and 5/. Avg. = averaged value.

aa	$^{4/s}$pp-aa	$^{5/s}$pp-aa	$^{aa/s}$M2$_e$	$^{aa/d}$M2$_e$	$^{aa/s}$M2$_e$	$^{aa/s}$E$_t$	$^{aa/d}$E$_t$	$^{aa/s}$E$_t$
			1r		mr	1r		mr
G	0.14	1.06	0.24	–0.03	–0.03	0.25	0.14	0.09
A	0.09	0.74	0.23	0.02	0.14	–0.01	0.09	–0.10
V	0.12	0.51	0.03	0.07	0.14	–0.04	0.14	0.13
L	–0.07	1.16	0.35	–0.03	0.28	0.18	0.09	0.10
I	–0.02	0.42	0.06		0.02	0.11		0.24
S	0.12	1.29	0.02		0.14	0.35		0.54
T	0.13	1.22	0.10		–0.02	0.39		0.27
C	0.04	0.63	0.11		–0.09	0.37		0.02
M	0.64	0.23	0.14		0.13	0.35		0.35
K	0.61	–0.07	–0.08	–0.05	0.11	0.59	0.29	0.50
R	–1.07	–0.44	–0.14	0.04	0.18	0.24	0.14	0.44
H	0.01	1.71	0.35	0.05	0.30	0.90	0.49	0.59
D	0.41	5.19	–0.25	–0.13	–0.26	1.02	0.58	0.83
E	0.01	3.57	0.10	–0.04	0.10	0.78	0.47	0.65
N	0.27	1.26	0.16		0.17	0.30		0.29
Q	0.05	1.46	0.09		0.17	0.44		0.46
P	0.03	–0.12	0.15		0.22	–0.11		0.15
F	0.04	–0.13	0.41		0.11	0.28		0.50
Y	–0.23	–0.29	0.24		0.24	0.14		0.48
W	0.21	0.35	0.34	–0.09	0.08	0.05	0.03	0.22
Avg.	0.08 ± 0.34	0.99 ± 1.34	0.13 ± 0.17	–0.02 ± 0.07	0.11 ± 0.13	0.33 ± 0.30	0.25 ± 0.20	0.34 ± 0.24

Table S 5: Estimated binding energy differences (ΔEBEs) between groups of assembled TMDs. The ΔEBEs are averaged over groups of poly-peptides (pp's), which each consists of one of the 20 amino acids (aa), used in the docking: GA, VLI, ST, CM, KRH, DE, NQP, FYW. Each of the difference of each pp is calculated between (i) the EBEs of CG models of bundles in which the to-be-docked pp and pp's are modeled as ideal helices (i, see also in Figure 3) and (ii) the EBEs when modeled with TMDs and pp's for which the Cα atoms are adopted to the same coordinates as the experimental structures (Cα, see also in Figure 3). Details of the calculations and the notations are given in Supplementary Table S4.

aa-p	$^{4/s}$p-aa	$^{5/s}$p-aa	$^{aa\text{-}s}M2_e$	$^{aa\text{-}d}M2_e$	$^{aa\text{-}s}M2_e$	$^{aa\text{-}s}E_{e/t}$	$^{aa\text{-}d}E_{e/t}$	$^{aa\text{-}s}E_{e/t}$
			1r		mr	1r		mr
G	0.14	1.06	0.24	–0.03	–0.03	0.25	0.14	0.09
A	0.09	0.74	0.23	0.02	0.14	–0.01	0.09	–0.10
Avg.	0.12 ± 0.03	0.90 ± 0.23	0.24 ± 0.01	0.00 ± 0.04	0.06 ± 0.12	0.12 ± 0.18	0.11 ± 0.04	0.00 ± 0.13
V	0.12	0.51	0.03	0.07	0.14	–0.04	0.14	0.13
L	–0.07	1.16	0.35	–0.03	0.28	0.18	0.09	0.10
I	–0.02	0.42	0.06		0.02	0.11		0.24
Avg.	0.01 ± 0.10	0.70 ± 0.40	0.15 ± 0.18	0.02 ± 0.07	0.14 ± 0.13	0.08 ± 0.11	0.12 ± 0.03	0.16 ± 0.07
S	0.12	1.29	0.02		0.14	0.35		0.54
T	0.13	1.22	0.10		–0.02	0.39		0.27
Avg.	0.12 ± 0.00	1.26 ± 0.05	0.06 ± 0.06		0.06 ± 0.11	0.37 ± 0.03		0.40 ± 0.20
C	0.04	0.63	0.11		–0.09	0.37		0.02
M	0.64	0.23	0.14		0.13	0.35		0.35
Avg.	0.34 ± 0.42	0.43 ± 0.28	0.12 ± 0.02		0.02 ± 0.15	0.36 ± 0.04		0.19 ± 0.23
K	0.61	–0.07	–0.08	–0.05	0.11	0.59	0.29	0.50
R	–1.07	–0.44	–0.14	0.04	0.18	0.24	0.14	0.44
H	0.01	1.71	0.35	0.05	0.30	0.90	0.49	0.59
Avg.	-0.15 ± 0.85	0.40 ± 1.15	0.04 ± 0.26	0.01 ± 0.05	0.20 ± 0.09	0.57 ± 0.33	0.31 ± 0.18	0.51 ± 0.08
D	0.41	5.19	–0.25	–0.13	–0.26	1.02	0.58	0.83
E	0.01	3.57	0.10	–0.04	0.10	0.78	0.47	0.65
Avg.	0.21 ± 0.28	4.38 ± 1.15	0.07 ± 0.25	-0.09 ± 0.07	-0.08 ± 0.26	0.90 ± 0.17	0.52 ± 0.07	0.74 ± 0.13
N	0.27	1.26	0.16		0.17	0.30		0.29
Q	0.05	1.46	0.09		0.17	0.44		0.46
P	0.03	–0.12	0.15		0.22	–0.11		0.15
Avg.	0.12 ± 0.13	0.87 ± 0.86	0.14 ± 0.04		0.19 ± 0.03	0.21 ± 0.29		0.30 ± 0.15
F	0.04	–0.13	0.41		0.11	0.28		0.50
Y	–0.23	–0.29	0.24		0.24	0.14		0.48
W	0.21	0.35	0.34	–0.09	0.08	0.05	0.03	0.22
Avg.	0.01 ± 0.22	–0.02 ± 0.33	0.33 ± 0.08		0.14 ± 0.09	0.16 ± 0.11		0.40 ± 0.15

Supplementary Table S5 (cont.)

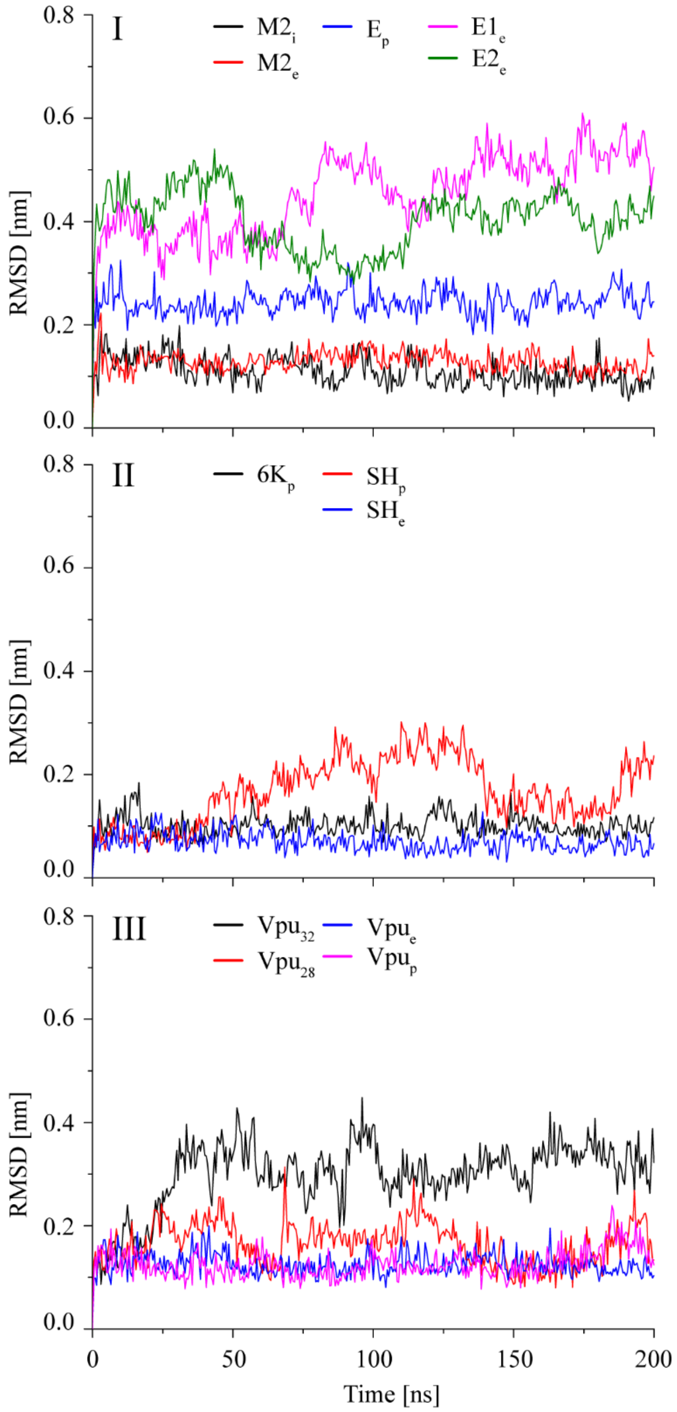

Figure S1: Root mean square displacement values over Cα atoms of each structure calculated every 500 ps. T he peptides simulated are (I) M2$_i$ (black line) M2$_e$ (red line), E$_p$ (blue line), E1$_e$ and E2$_e$ (pink and green lines, respectively with 1 and 2 marking the first and second E protein in the simulation box); (II) 6K$_p$ (black line), SH$_p$ (red line) and SH$_e$ (blue line); (III) Vpu$_{32}$ (black line), Vpu$_{28}$ (red line), Vpu$_e$ (blue line), Vpu$_p$ (pink line).

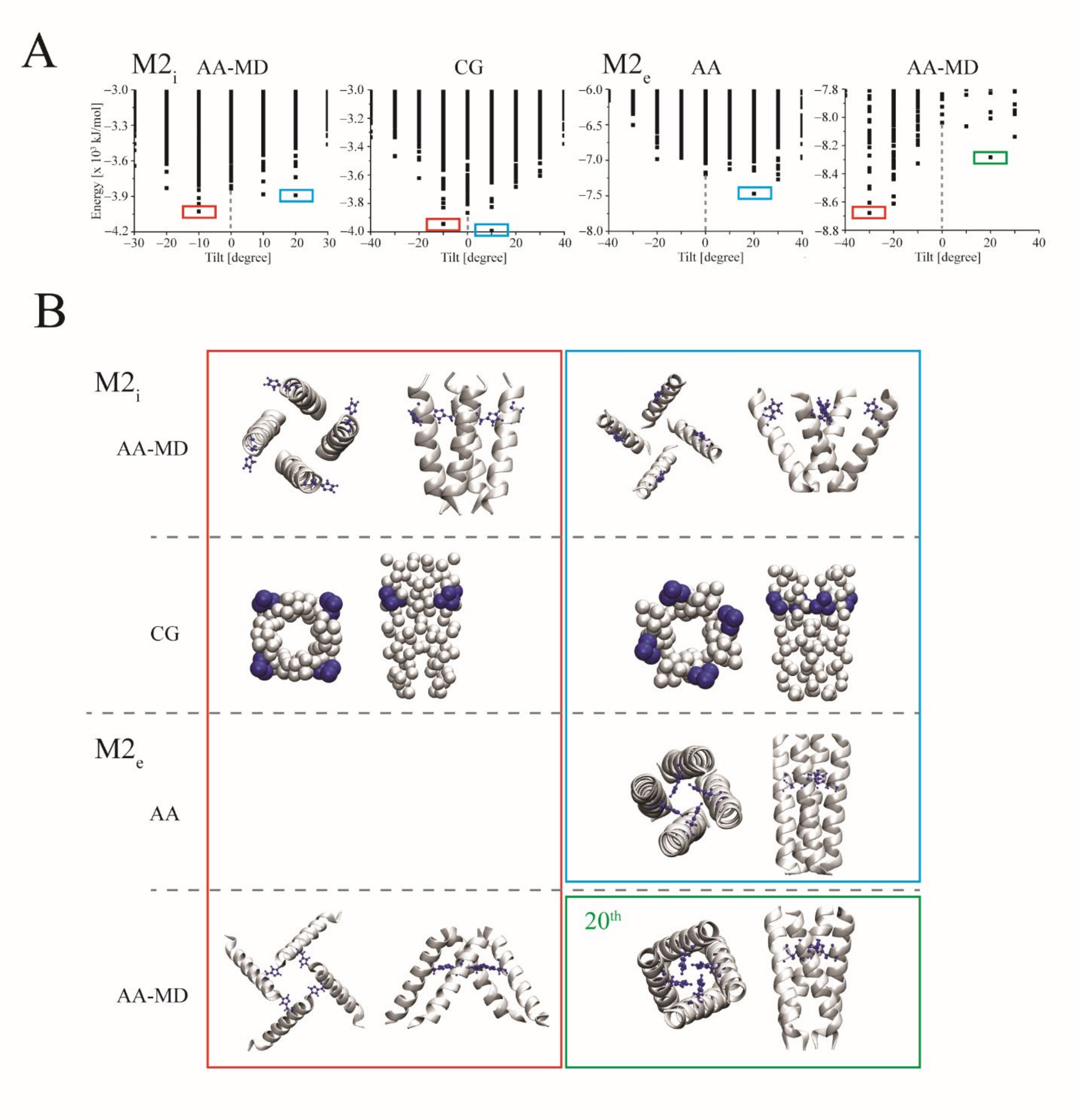

Supplementary Figure S2

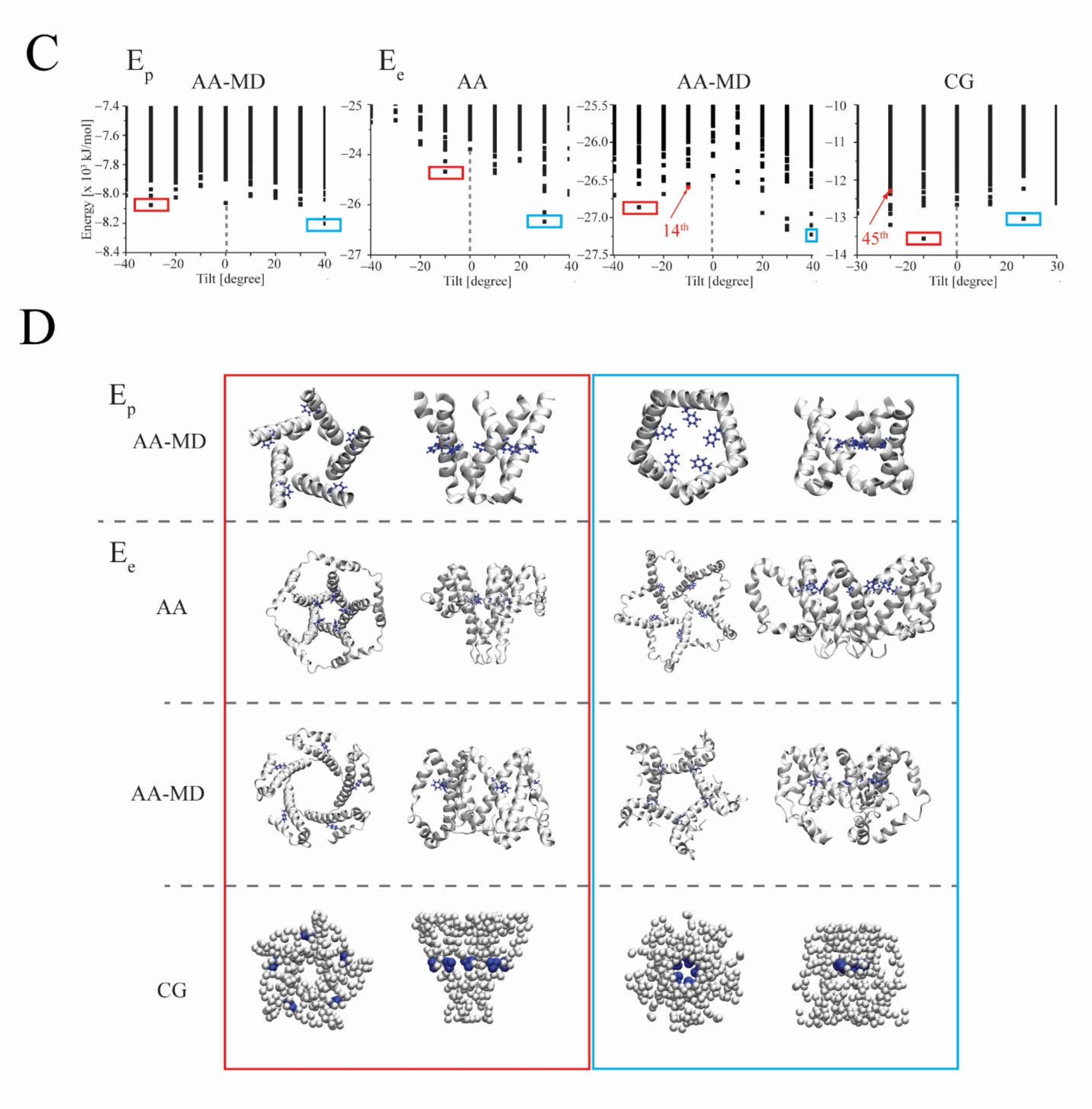

Supplementary Figure S2 (cont.)

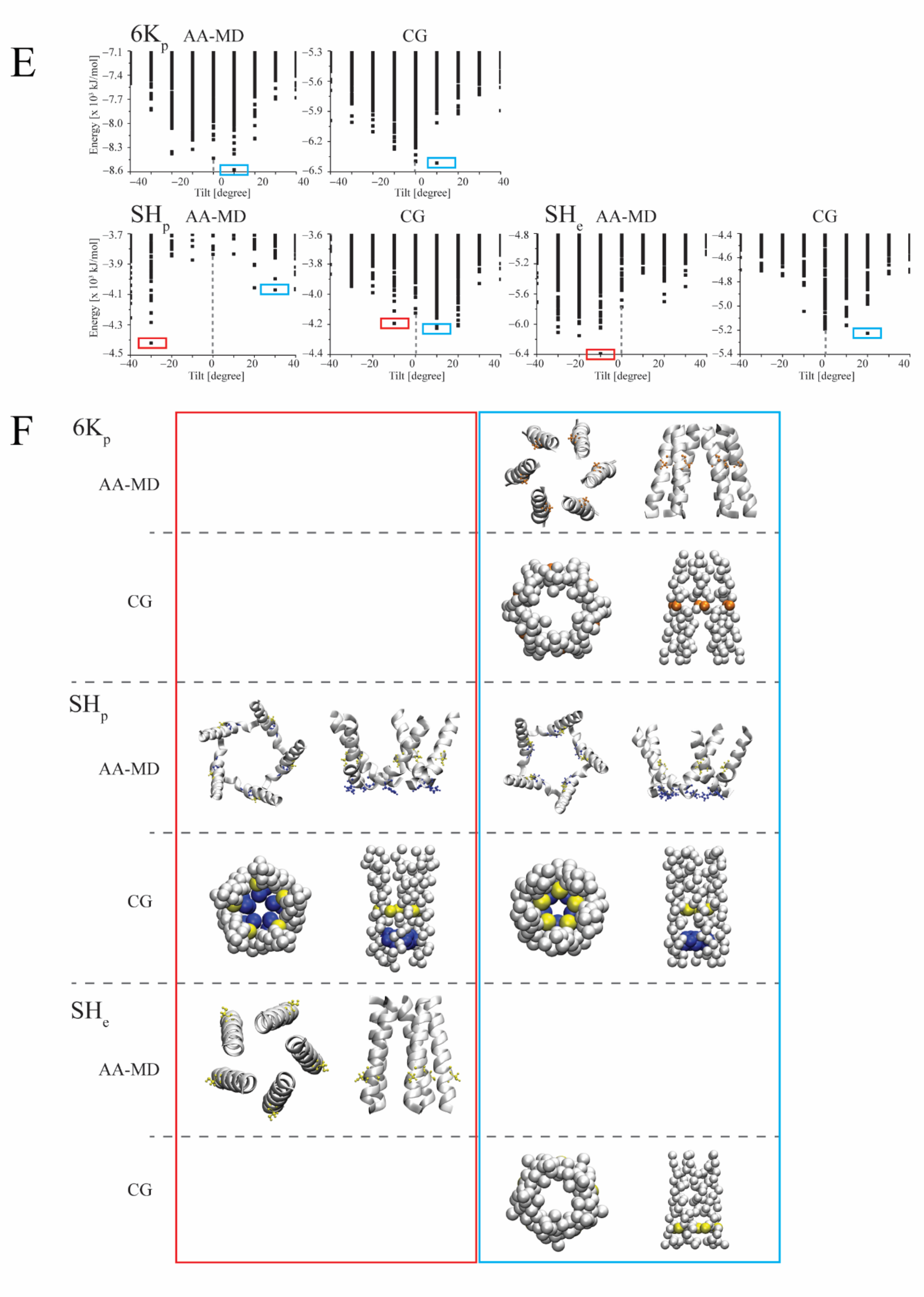

Supplementary Figure S2 (cont.)

Figure S2: Ranking of the assembled bundles based on estimated binding energies (EBEs) over tilt from applying S1 protocol and visualization of the structures. (A) EBEs over tilt of assembled tetrameric $M2_i$ and $M2_e$. The green box marks a minimum value for which handedness and orientation of the marker-residue (mr) matches the experimental position. (B) Structural models of the respective bundles $M2_i$ and $M2_e$. The side chains mark residue H37 either in dark blue ball and stick modus or respective spheres for the CG models. (C) EBEs over tilt of assembled pentameric E_e and E_p bundles. Bundles in which the mr F26 is pointing into the pore are indicated by their rank (red numbers and arrows). (D) Structural models of the respective bundles E_e and E_p. Side chains mark residue F26 in dark blue for E. (E) EBEs of assembled hexameric $6K_p$, pentameric SH_p and SH_e bundles over the tilt. (F) Structural models of the respective bundles $6K_p$, SH_p and SH_e. Side chains mark the following residues: A28 (orange) for 6K, H22 (blue), S29 (yellow) for SH_p and SH_e, respectively.

The structural models used are all atom models with TMDs from a MD simulation (AA-MD), for coarse-grained models (CG), as well as for all atom models (AA). The subscripts indicate the use of: i = ideal helices; e = structures or sequence used from experimental sources; p = ideal structures from sequences of amino acids predicted to form a helical TMD. The grey dashed line marks the energy value for zero tilt. The small blue and red boxes in the plots mark minimum energies for energies with tilts larger and lower, respectively, than zero tilt. Structural models of the respective bundles are surrounded by red (tilt < 0 tilt) and blue boxes (tilt > 0) in top (from N to C) and side view (cytoplasmic side pointing downwards). The helix-backbone is shown in grey cartoon mode. Amino acid side chains are shown either in ball-and-stick model for AA models or spheres for CG models.

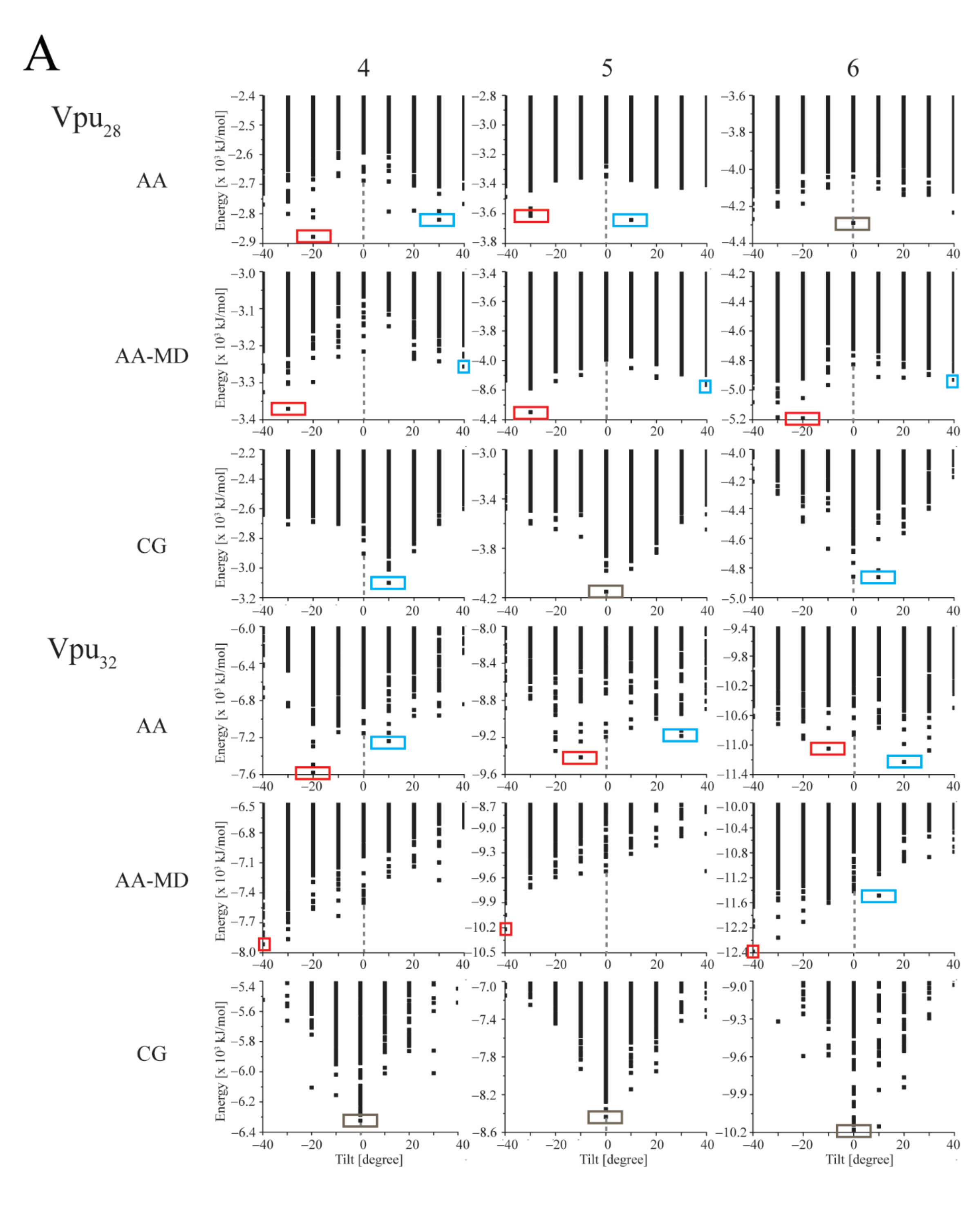

Supplementary Figure S3

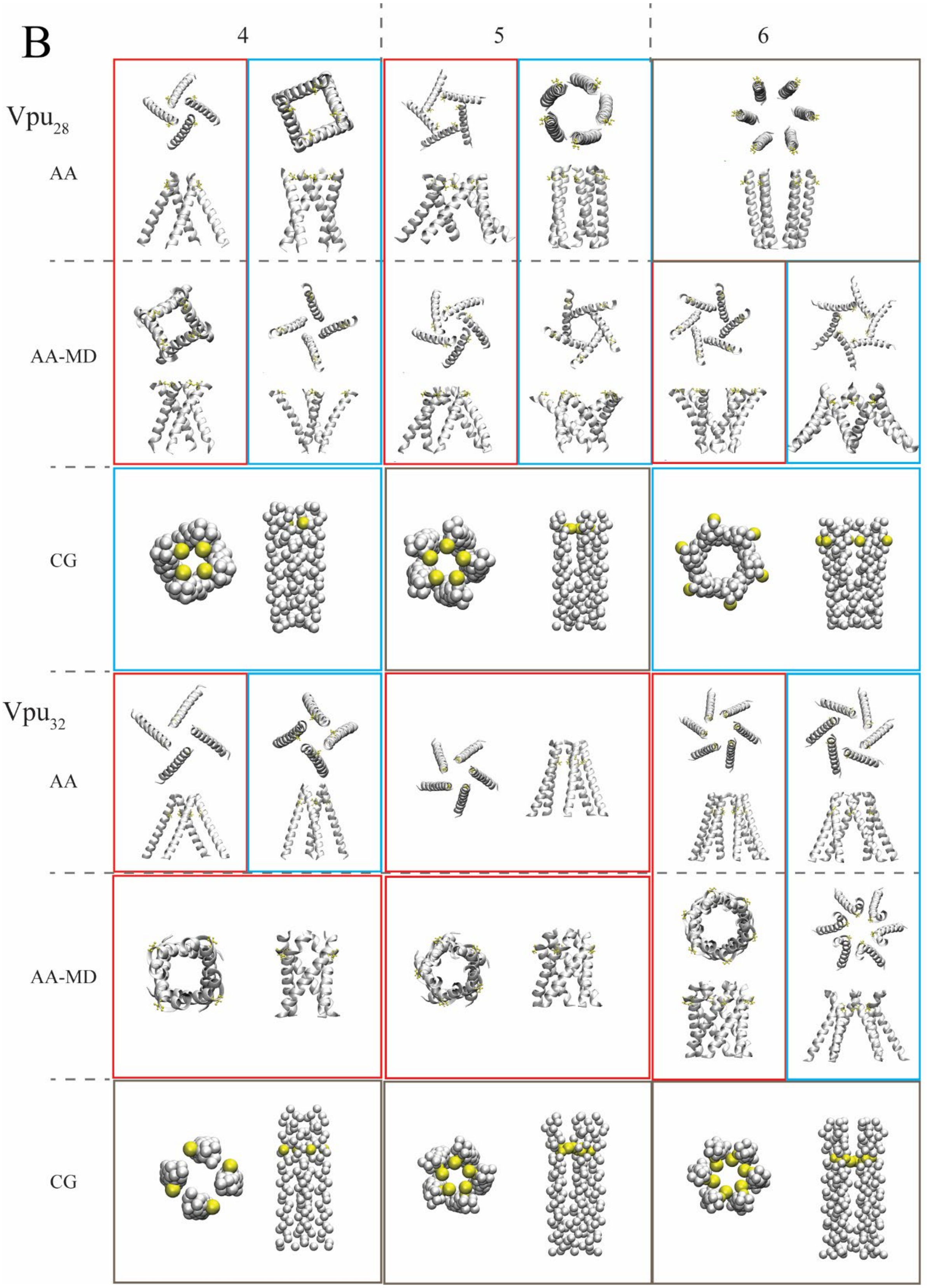

Supplementary Figure S3 (cont.)

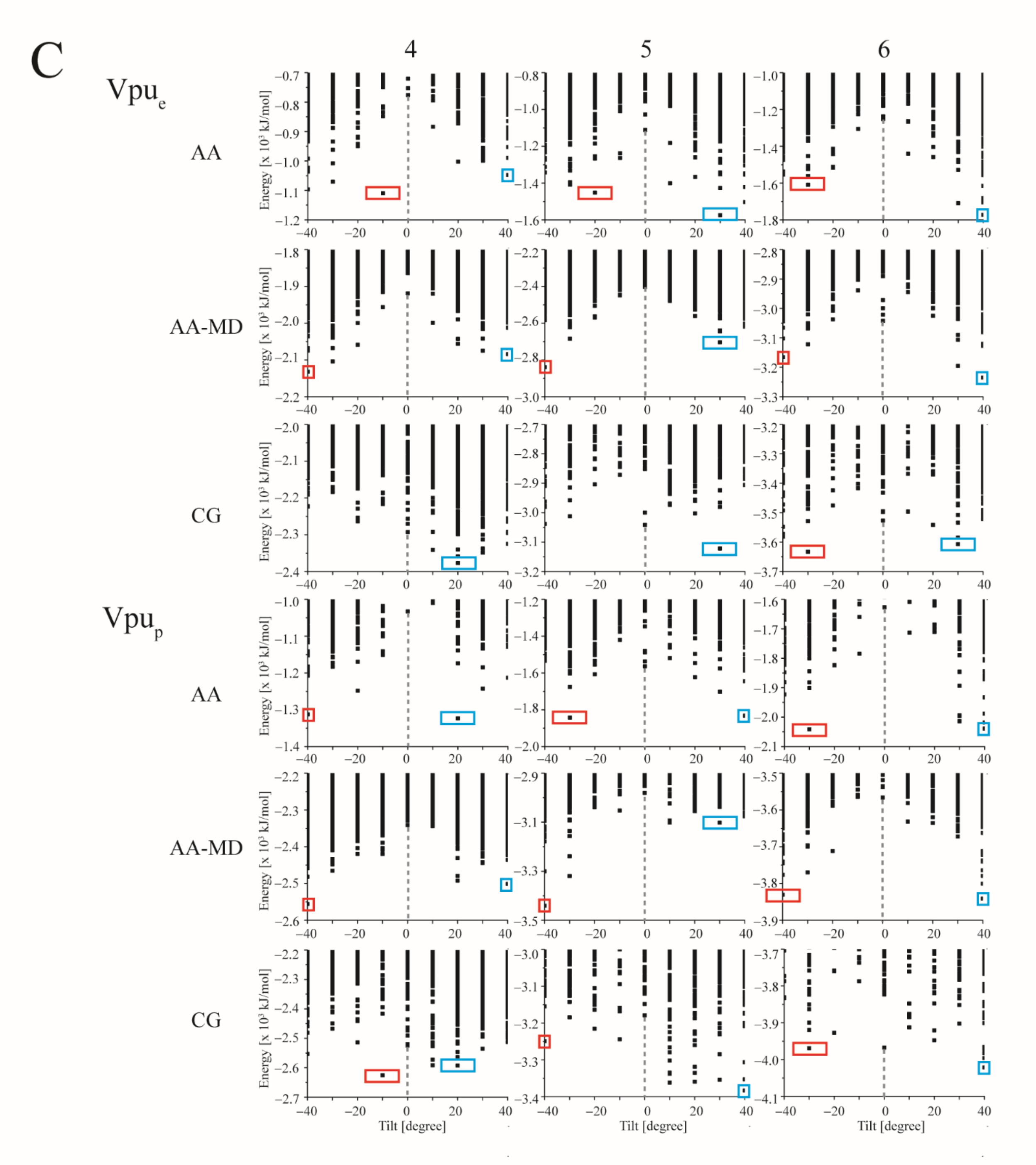

Supplementary Figure S3 (cont.)

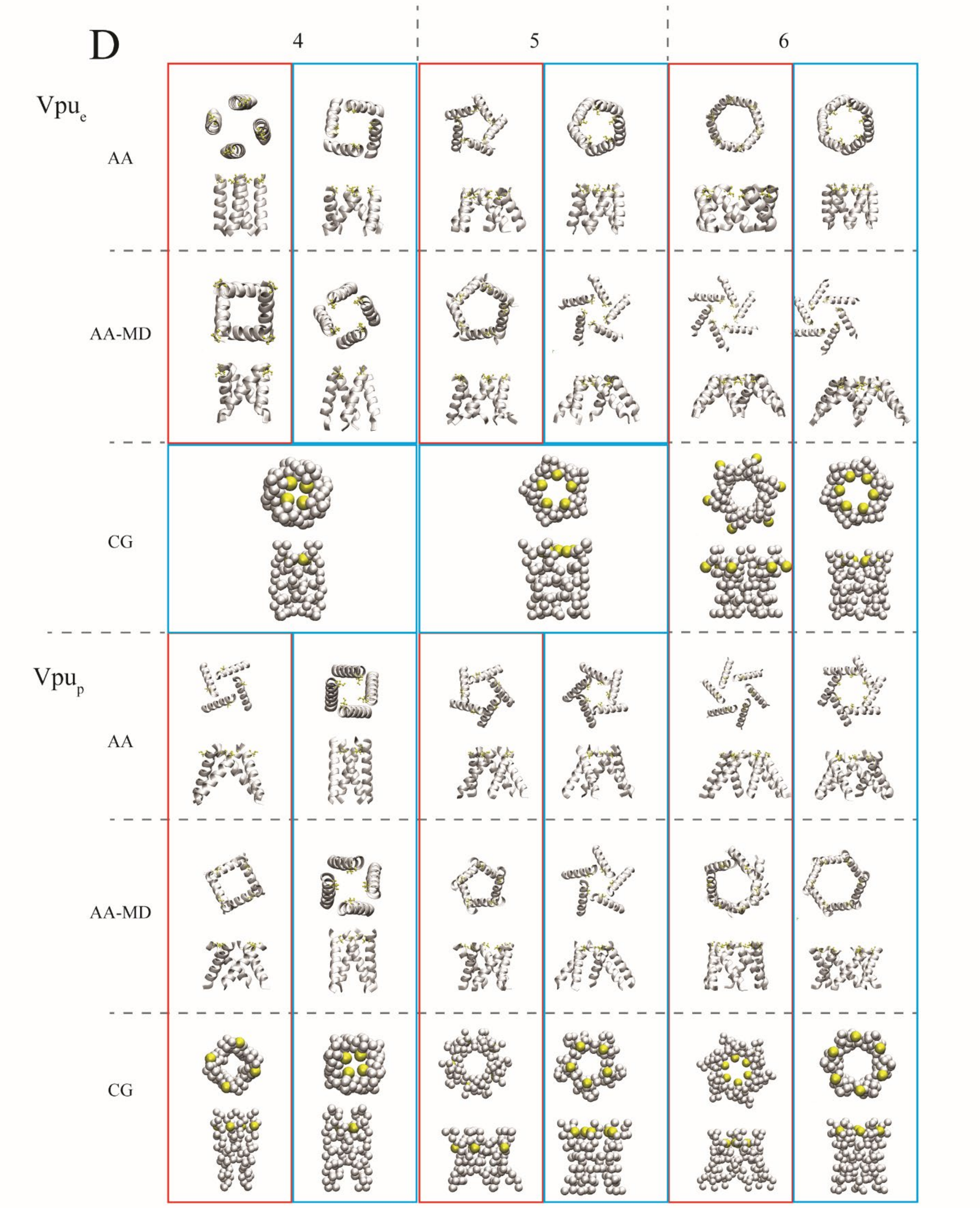

Supplementary Figure S3 (cont.)

Figure S3: Ranking of the assembled bundles based on estimated binding energies (EBEs) over tilt from applying S1 protocol and visualization of the structures of Vpu. (A) EBEs over tilt for assembled tetra-, penta- and hexameric Vpu_{28} and Vpu_{32} bundles. (B) Structural models of the respective bundles Vpu_{28} and Vpu_{32}. The side chains mark the following mr S24 (yellow). (C) EBEs of assembled bundles Vpu_e and Vpu_p. (D) Structural models of the respective bundles Vpu_e and Vpu_p.

The structural models used are all atom models (AA), models with TMDs from a MD simulation (AA-MD), and coarse-grained models (CG). The subscripts indicate the use of: 32 and 28 = numbers of amino acids modeling ideal TMDs; e = structures or sequence used from experimental sources; p = ideal structures from sequences of amino acids predicted to form a helical TMD. The 'oligomers' are sorted according to the number of TMDs forming the bundle. The grey dashed line marks the minimum estimated binding energy (EBE) for zero tilt. The small blue and red boxes in the plots mark EBEs for structures with tilts larger and lower, respectively, than zero tilt. Structural models of the respective bundles are surrounded by red (tilt < 0 tilt) and blue boxes (tilt > 0) in top (from N to C) and side view (cytoplasmic side pointing downwards). The helix-backbone is shown in grey cartoon mode. The helix-backbone is shown in grey cartoon mode. Amino acid side chains are shown either in ball-and-stick mode for the AA models or as spheres for the CG models.

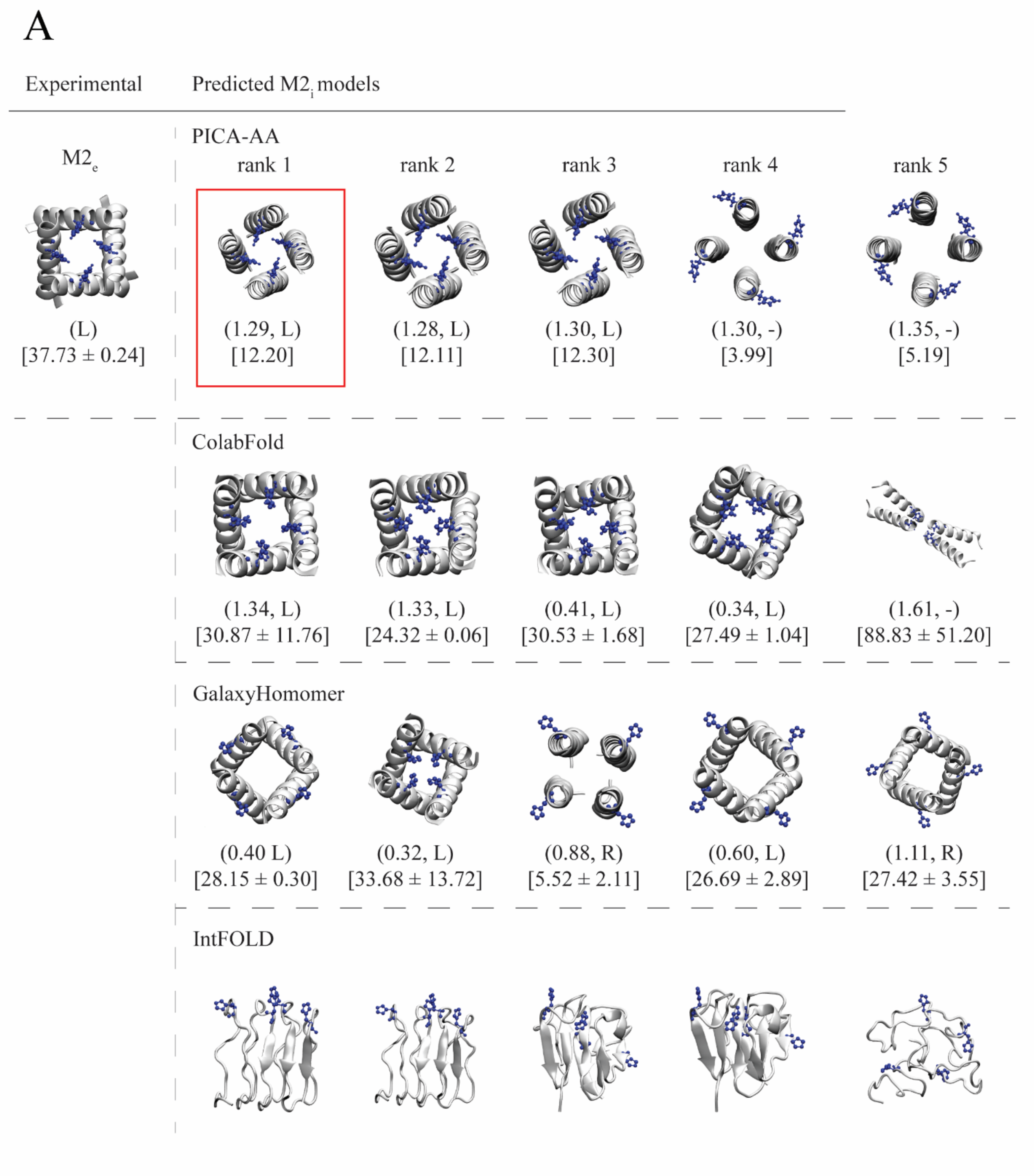

Supplementary Figure S4

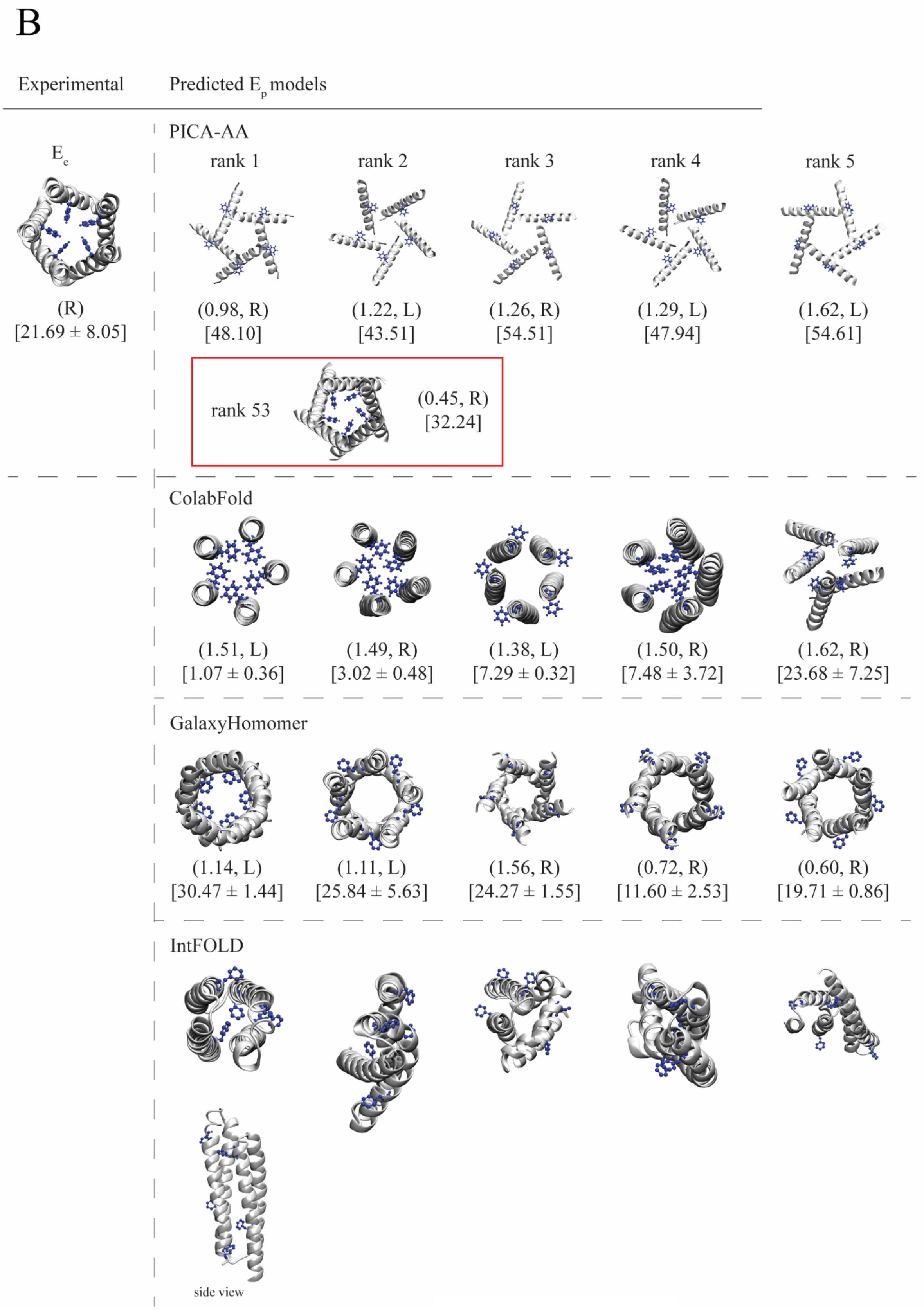

Supplementary Figure S4 (cont.)

Figure S4: Comparison of assembled tetrameric M2, and pentameric E protein. All atom (AA) models using PICA and an available software ColabFold (DOI: 10.1038/ s41592-022-01488-1, reference [66] in the manuscript), GalaxyHomomer (DOI: 10.1093/ nar/gkx246, [67] in manuscript) and IntFold (DOI: 10.1093/nar/gkz322, [68] in manuscript) with the existing crystal structures of M2 (PDB ID: 1NYJ) and E protein (PDB ID: 5X29). For PICA all atom (AA) models of $M2_e$ and E_e are used using the same amino acid sequence as for $M2_i$ and E_p, respectively. For all other server's amino acid sequences as for $M2_i$ and E_p are used. Numbers in brackets represent the root mean square deviation (RMSD) values in nm of the structures with reference to the experimental structures. Letters R and L refer to right- and left-handed bundles, respectively, of the crystal structures. Numbers in squared brackets represent the tilt angles (°), given as average over the TMDs of a bundle with standard deviation. The numbers for all models are presented in in Table 2). The best models of PICA compared with the experimental structure are highlighted in a red box.

The structural models are shown with their backbone (grey) with the marker residues H37 for M2 and F26 for E protein in ball-stick modus in blue. The subscripts indicate the use of: i = ideal helix used; e = sequence as in experimental study reported in literature or experimental structure used for which PDB ID is available; p = ideal structures from sequences of amino acids predicted to form a helical TMD.

A

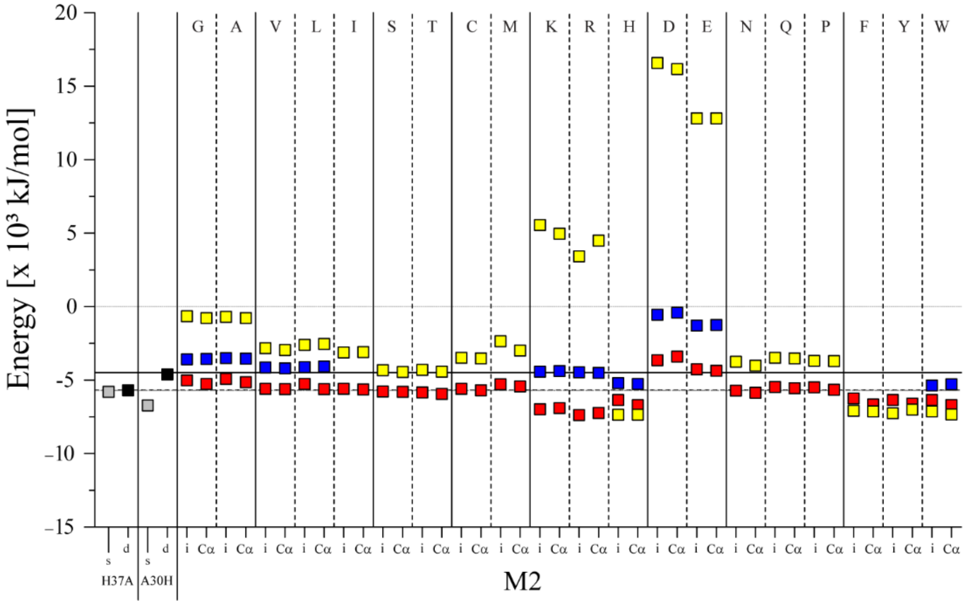

B

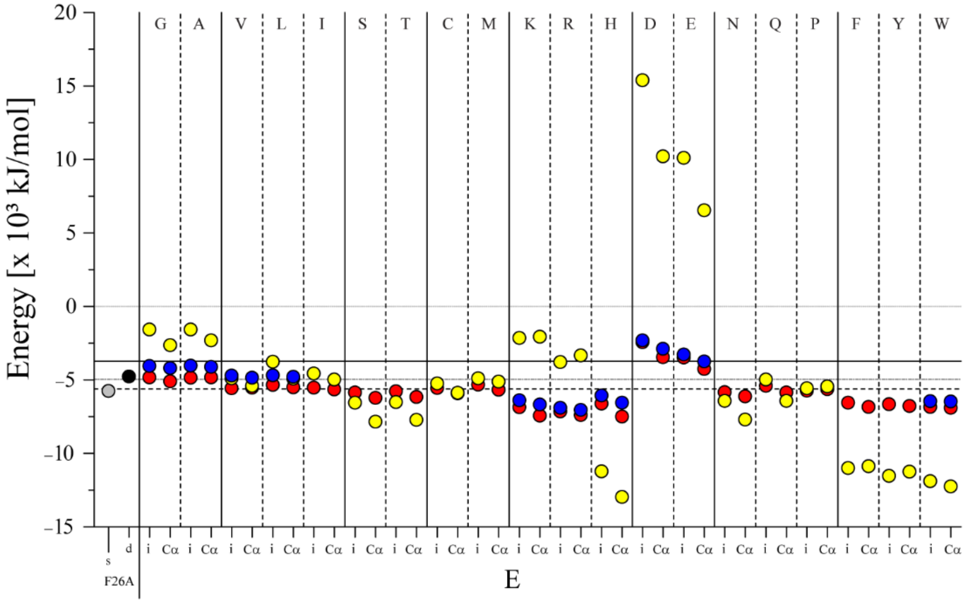

Supplementary Figure S5

Figure S5: Ranked estimated binding energies (EBEs) of single helical poly-peptides (pp's) assembled with target peptides as well as with itself for target peptides for which the marker-residues (mr) are pore-lining. Each of the single pp's generated for each of the 20 amino acids is docked to (A) M2 (PDB ID: 1NYJ) with the EBE values marked as squares and (B) to a truncated version of E (PDB ID: 5X29), E_t, marking the EBEs with circles. The values are calculated for CG-models of pp's as either ideal helices (i, see also in Figure 3) or those with helices for which the Cα atoms adopt the same coordinates as the experimental structures (Cα, see also in Figure 3) being assembled with oligomeric M2 or E bundles. For both plots, individual single pp's are assembled synchronously (s-screening, s) with three TMDs (1+3) of M2 or four TMDs of E (1+4) (red), as well as 4 (in (A)) and 5 pp's (in (B)) with themselves (yellow). In addition, the pp's are docked using dimeric docking (d-screening, d) in which three or four of the TMDs of M2 and E, respectively, were preformed/taken from the respective crystal structures and docked with the p-peptide (blue). Docking of mutants, H37A and A30H for M2 and F26A for E, are shown for s-screening (grey) and d-screening (black). The pp's are either used as ideal helices, marked with i, or as helices for which the Cα atoms adopt the same coordinates as the experimental structures, marked with Cα. Reference lines are marking the EBEs of (i) the crystal structures of $M2_e$ and E_t (black lines), (ii) the 1r redocked $M2_e$ ad E_t structures using s-docking, ^sM2_e and ^sE_t, (black dashed line), and (iii) the 1r redocked $M2_e$ and E_t structures using d-docking, ^dM2_e and ^dE_t (grey line). For EBEs see Supplementary Table S2.
